# Fetal Mesenchymal Stromal Cells Differentiating towards Chondrocytes Acquire a Gene Expression Profile Resembling Human Growth Plate Cartilage

**DOI:** 10.1371/journal.pone.0044561

**Published:** 2012-11-05

**Authors:** Sandy A. van Gool, Joyce A. M. Emons, Jeroen C. H. Leijten, Eva Decker, Carsten Sticht, Johannes C. van Houwelingen, Jelle J. Goeman, Carin Kleijburg, Sicco A. Scherjon, Norbert Gretz, Jan Maarten Wit, Gudrun Rappold, Janine N. Post, Marcel Karperien

**Affiliations:** 1 Department of Pediatrics, Leiden University Medical Center, Leiden, The Netherlands; 2 Department of Developmental BioEngineering, MIRA Institute for Biomedical Technology and Technical Medicine, University of Twente, Enschede, The Netherlands; 3 Department of Human Molecular Genetics, University of Heidelberg, Heidelberg, Germany; 4 Medical Research Center, Medical Faculty Mannheim, University of Heidelberg, Heidelberg, Germany; 5 Department of Medical Statistics and Bioinformatics, Leiden University Medical Center, Leiden, The Netherlands; 6 Department of Obstetrics, Leiden University Medical Center, Leiden, The Netherlands; University of Maryland School of Medicine, United States of America

## Abstract

We used human fetal bone marrow-derived mesenchymal stromal cells (hfMSCs) differentiating towards chondrocytes as an alternative model for the human growth plate (GP). Our aims were to study gene expression patterns associated with chondrogenic differentiation to assess whether chondrocytes derived from hfMSCs are a suitable model for studying the development and maturation of the GP. hfMSCs efficiently formed hyaline cartilage in a pellet culture in the presence of TGFβ3 and BMP6. Microarray and principal component analysis were applied to study gene expression profiles during chondrogenic differentiation. A set of 232 genes was found to correlate with *in vitro* cartilage formation. Several identified genes are known to be involved in cartilage formation and validate the robustness of the differentiating hfMSC model. KEGG pathway analysis using the 232 genes revealed 9 significant signaling pathways correlated with cartilage formation. To determine the progression of growth plate cartilage formation, we compared the gene expression profile of differentiating hfMSCs with previously established expression profiles of epiphyseal GP cartilage. As differentiation towards chondrocytes proceeds, hfMSCs gradually obtain a gene expression profile resembling epiphyseal GP cartilage. We visualized the differences in gene expression profiles as protein interaction clusters and identified many protein clusters that are activated during the early chondrogenic differentiation of hfMSCs showing the potential of this system to study GP development.

## Introduction

Longitudinal bone growth is the result of a tightly orchestrated proliferation and differentiation program called endochondral ossification. In the epiphyseal growth plate, chondrocytes originating from mesenchymal stromal cells subsequently undergo proliferation, hypertrophic differentiation, and programmed cell death before being replaced by bone. At the time of puberty, growth first increases but at the end of puberty epiphyseal fusion and termination of growth occurs. Although estrogen has been identified as a key regulator of growth plate maturation and fusion [1], our knowledge of the molecular mechanisms underlying human growth regulation during puberty is limited. Gaining a detailed understanding of growth regulatory processes is essential to facilitate the development of novel strategies for the treatment of various growth disorders.

Commonly used animal models for studying growth plate regulation represent the human epiphyseal growth plate poorly. One of the most obvious differences is that rodent growth plates do not fuse at the end of sexual maturation [2], an important hallmark of human growth plate development. The shortcoming of the mouse model is furthermore demonstrated by the contrast between the marginally affected growth phenotype of the estrogen receptor alpha (ERα) knock out mouse (αERKO) [3] and the prominent growth phenotype of a male patient lacking functional ERα [4], which is characterized by the absence of epiphyseal fusion and continuation of growth into adulthood.

In order to elucidate the mechanisms involved in growth plate regulation and fusion, we need to employ different strategies. The most optimal strategy would be to investigate these processes in human growth plate specimens at different times during development. However, human growth plate specimens are difficult to obtain [5]. *In vitro* models such as chondrosarcoma cell lines or articular cartilage-derived chondrocyte cultures have limited differentiation capacity, are often difficult to maintain under laboratory conditions or tend to dedifferentiate [6], [7]. Furthermore, articular cartilage and growth plate cartilage have distinct functions and it is therefore debatable whether articular cartilage-derived chondrocytes are representative for epiphyseal growth plate chondrocytes.

Multipotent human mesenchymal stromal cells (hMSCs) are promising for studying chondrogenesis *in vitro*. hMSCs are an alternative cell source for articular cartilage reconstruction and for studying endochondral ossification as it occurs in the epiphyseal growth plate [8]. In addition, we found that MSCs in co-culture with chondrocytes contribute greatly to better cartilage generation in vitro [9]. In this study, we explored the cartilage forming capacity of human fetal (hf)MSCs in time. In addition, we explored whether differentiating hfMSCs may provide an *in vitro* model for the human growth plate. We have chosen human fetal bone marrow-derived MSC for their superior differentiation characteristics compared to adult bone marrow-derived MSCs [10]. Efficient cartilage formation was demonstrated by immunohistochemical analysis and gene expression profiling was applied to identify genetic pathways involved in the differentiation process. In addition, gene expression profiles of the differentiating hfMSCs were compared with global gene expression patterns of human articular and growth plate cartilage to assess whether differentiating hfMSCs represent either articular or growth plate chondrocytes.

## Results

### 2.1 Chondrogenically differentiating hfMSCs express typical markers of hyaline cartilage

#### 2.1.1. Evaluation of protein and mRNA expression

Two major types of hyaline cartilage can be distinguished, namely articular and epiphyseal cartilage. In order to serve as a model for the epiphyseal growth plate, differentiating hfMSCs should obtain a growth plate signature.

**Figure 1:**
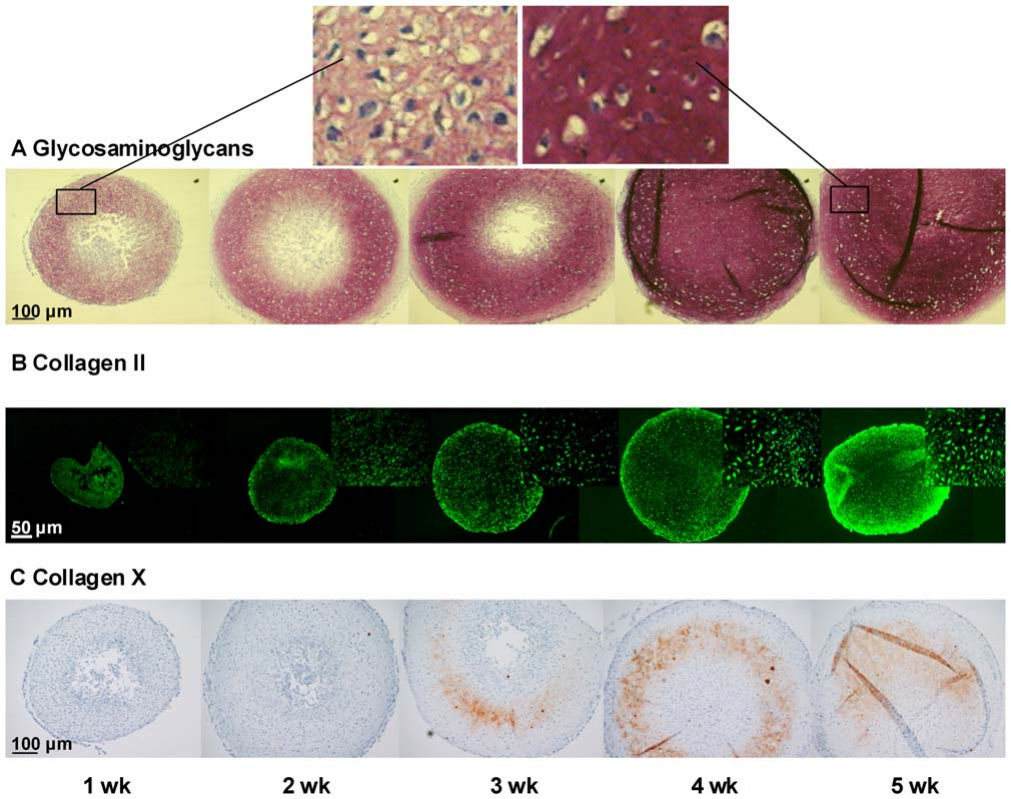
hfMSCs micromasses undergo chondrogenic differentiation. Expression of (A) glycosaminoglycans visualized by toluidine blue staining, nuclei are counterstained with haematoxylin, (B) collagen type II immunofluorescence, and (C) collagen type X immunohistochemistry (red-brown) during 5 weeks of chondrogenic differentiation of hfMSCs to chondrocytes. The top panel shows a magnification of the pellet cultures at week 1 and week 5 stained by toluidine blue demonstrating the change in cell morphology and the deposition of the extracellular matrix. The insets in panel B show higher magnifications of collagen type II positive chondrocytes.

Pellet cultures were used to induce chondrogenic differentiation of hfMSCs and samples were collected at 1, 2, 3, 4 and 5 weeks of culture. Immunohistological evaluation showed an increasing expression of cartilage markers with time and a gradual morphological change from stromal cells to mature and hypertrophic chondrocytes (Figure 1). The mean diameter of the pellets increased with time, as well as the amount of glycosaminoglycans, a major constituent of the cartilaginous extracellular matrix. Immunofluorescent staining for collagen type II demonstrated the presence of chondrocytes after 1 week of pellet culture. The expression of collagen type II increased over time. Hypertrophic chondrocytes were first detected after 3 weeks, as evidenced by immunohistochemical staining for collagen type X. These collagen type X positive cells were located in a discrete ring-like zone surrounded by collagen type II positive chondrocytes. In all stages of differentiation, the chondrogenic core of the pellets was surrounded by a thin layer of two to three undifferentiated cells (Figure 1). Matrix mineralization was not observed after 5 weeks of differentiation (data not shown), suggesting that the matrix was not ready for mineralization or that environmental stimuli necessary to induce this process were absent.

From each time point RNA was isolated and subjected to microarray analysis. Changes in gene expression of a subset of genes consisting of both established marker genes for chondrogenesis and differentially expressed genes identified by microarray analysis were validated using qPCR (Figure S1). In concordance with the observations of immunohistological markers of chondrogenesis, microarray data and qPCR showed time-dependent increases in the expression of the cartilage markers *COL2A1*, ‘*COL10A1*, *SOX9*, and *ACAN* mRNA.

Previously, we have shown that chondrogenic differentiation of MSCs results in a growth plate (GP-) like rather than an articular phenotype [11]. In this study, we compared the gene expression profiles of human articular cartilage to human growth plate cartilage of the same donors. Global gene expression microarray analysis showed that the Wnt antagonist DKK1 and FRZB and the BMP antagonist GREM1 are highly expressed in articular cartilage as compared to growth plate cartilage. In contrast, PANX3, EPYC, WNT11 and LEF1 are highly expressed in growth plate cartilage as compared to articular cartilage. To investigate whether the differentiation of the hfMSCs in the chondrogenic lineage results in expression of markers specifically found in the GP vs AC, we selected genes that are significantly differentially expressed in these two types of hyaline cartilage; for GP: *PANX3*, *EPYC*, *WNT11*, *LEF1*, and for AC: *GREM1*, *FRZB*, *DKK1*.

**Figure 2:**
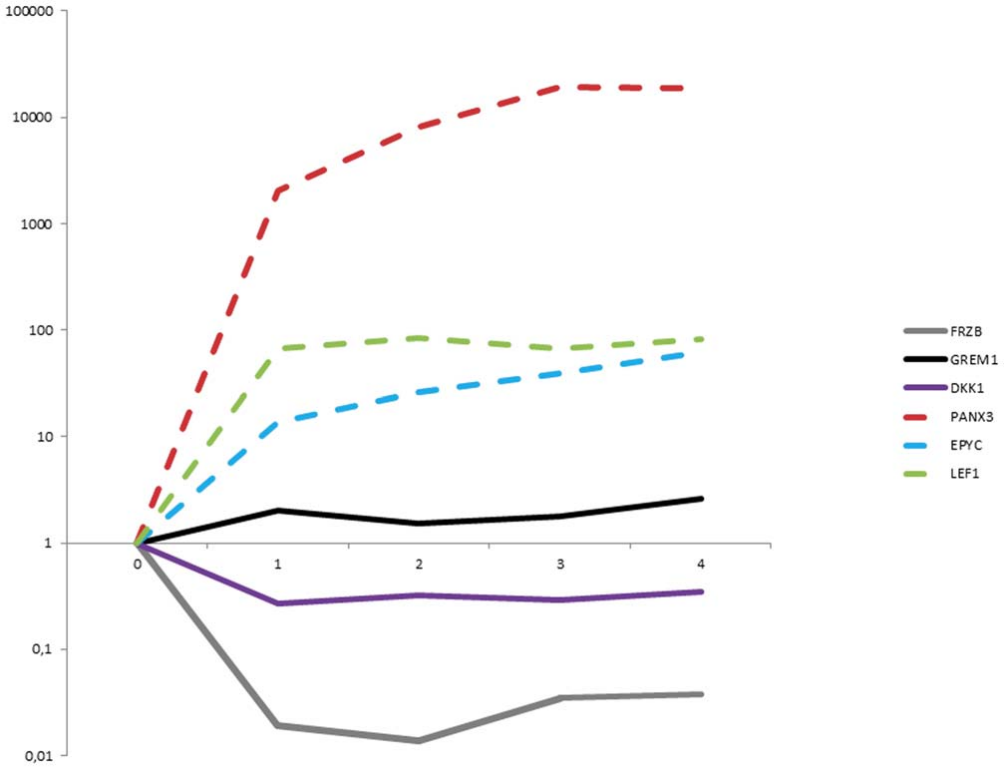
Increase in growth plate enriched genes and decrease of articular cartilage enriched genes in time. Expression of growth plate enriched genes *PANX3, EPYC, LEF1* and articular cartilage enriched genes *DKK1, FRZB, GREM1* during 4 weeks of chondrogenic differentiation of hfMSCs. The y-axis (left) indicates the qPCR results as normalized fold expression on a log-scale. The x-axis (right) indicates the time in weeks. Fold changes are calculated from qPCR data expressed as delta delta CT values corrected for the housekeeping gene *GAPDH*.

We observed a sharp increase in the expression of *PANX3*, *EPYC* and *LEF1* (GP enriched genes) in the differentiating MSCs while the AC enriched genes *FRZB*, and *DKK1* showed a marked decrease in their expression over time (figure 2). The gene expression of *GREM1* hardly changed over time. This indicates that the cells acquire a GP-like expression profile early during the differentiation.

#### 2.1.2. Principal component analysis and KEGG pathway analysis identifies 9 pathways associated with chondrogenic differentiation

**Figure 3:**
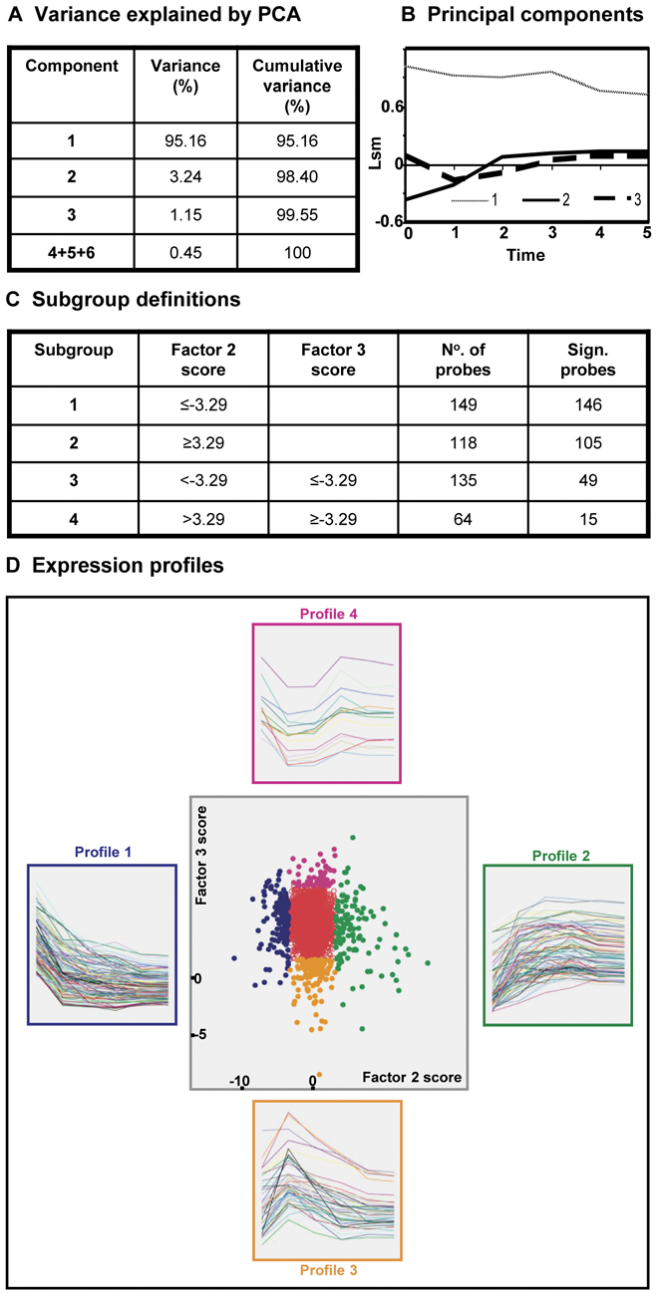
Analysis of changes in gene expression by microarray. Gene selection based on principal component analysis. A) variance explained by components 1–6 from principal component analysis. B) principal components 1, 2, and 3 as expression profiles. C) selection of probes based on their factor 2 and 3 scores. D) scatterplot view of gene data in respect to their correlation (factor score) to principal components 2 and 3. Subgroups 1, 2, 3, and 4 are represented by blue, green, yellow, and pink dots, respectively. Side-placed graphs depict the gene expression profiles for genes found in the four subgroups.

The sequential changes that occur during chondrogenic differentiation in the hfMSC model were studied with bioinformatics analysis of the microarray data. Using principal component analysis, three components were found to explain 99.6% of the variance within our dataset (figure 3A). The factor loadings in figure 3B show that component 1 describes a general level of gene expression, as expected. Component 2 shows to what extent gene expression changed with time during chondrogenic differentiation and component 3 signifies there was an additional, short-term elevation or dip in expression around 2 to 3 weeks of differentiation. Since components 2 and 3 were most likely to contain genes associated with the loss of stromal cell characteristics or the gain of a chondrocyte phenotype, we focused on those components.

Using the ≥3.29 cut-off in combination with a 5% significance test, we distinguished four subgroups of probes. The precise definitions and the resulting numbers of these subgroups are given in figure 3C. The scatter plot in figure 3D illustrates that the numbers of probes in subgroups 1 and 2 are much larger than the 9 probes (0.05%) that would have been expected under purely random selection. Moreover, in these two subgroups nearly all probes in the first selection are significant at the 5% level, suggesting that the number of false discoveries in these two groups is quite small. More noise is presumably present in the smaller subgroups 3 and 4 based on factor 3 scores.

The profiles of the selected probes demonstrate that subgroup 1 containing the largest number of probes (n = 146) describes a peak of expression on t_0_ (week 0) followed by a decrease in expression thereafter. In contrast, the second largest subgroup of probes (n = 105) in profile 2 demonstrates increasing expression levels from t_0_ onward. The smaller subgroups 3 and 4 demonstrate lower levels of expression with profile 3 (n = 49) showing a short-term increase in expression at t_1_ (week 1) followed by decreases thereafter and profile 4 (n = 15) displaying a short-term expression dip between t_1_-t_2_ (week 1- week 2).

**Figure 4:**
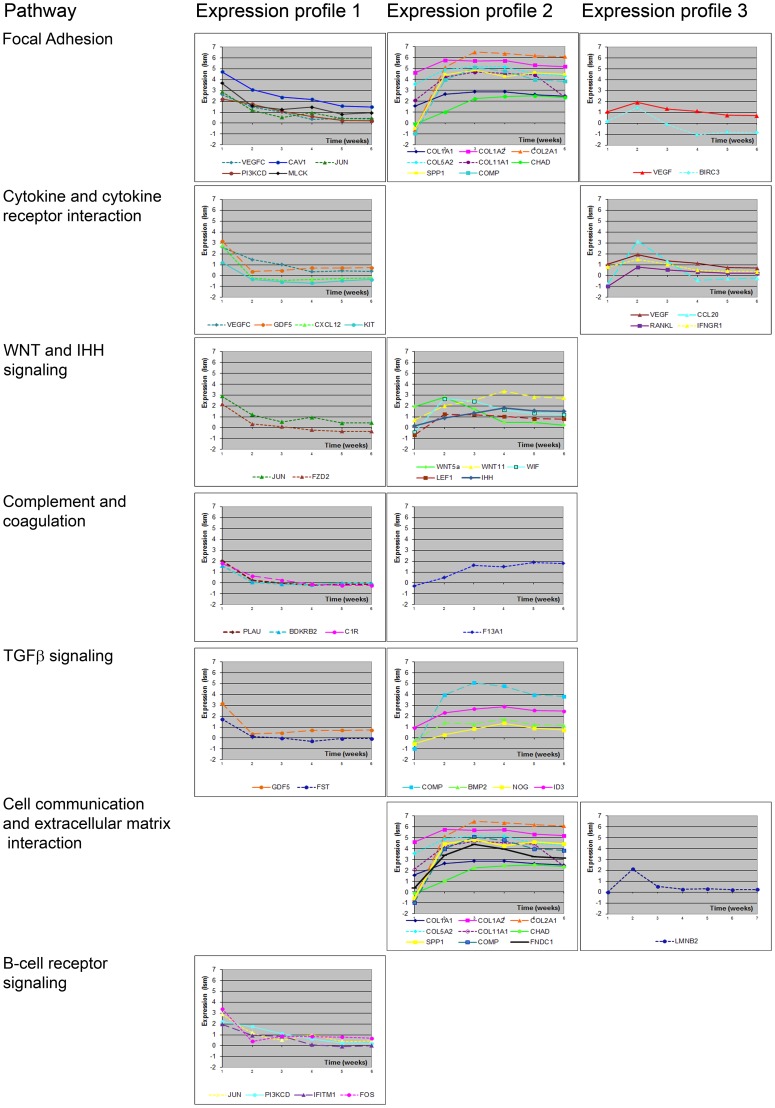
Gene expression of genes by KEGG signaling pathway. KEGG signaling pathways significantly associated with chondrogenic differentiation of hfMSCs. For each pathway, genes showing the same distinct expression profile during 5 weeks of chondrogenic differentiation are depicted as groups.

A total of 83 out of 315 probes could not be annotated and was discarded from further analysis. The remaining 232 probes that could be matched to genes (table S1) were used to identify 9 KEGG pathways that were significantly associated with chondrogenic differentiation and contained 39 genes (figure 4). Some genes were present solely in one pathway (n = 23), but others were found in 2 (n = 6) or 3 (n = 10) pathways (Figure 4). Three functional groups of genes were recognized: 1) growth factor (GF) and GF-related genes; 2) genes associated with the extracellular matrix; and 3) genes associated with signal transduction, cell cycle, and cell survival. In table S2, we have listed the top hits of upregulated and down-regulated genes (with a cut-off of at least 3.29-fold change) as identified by the microarray analysis at week 5 compared to undifferentiated hfMSCs.

#### 2.1.3 Network analysis of significantly changed genes identifies many chondrogenesis associated clusters

**Figure 5:**
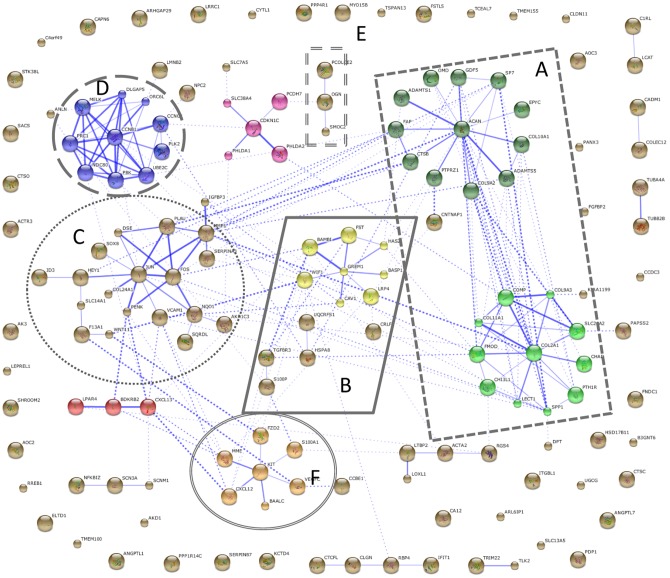
Protein interactions of significantly changed genes after 5 weeks of chondrogenic differentiation. Analysis of protein interactions of all genes that were ≥3.29-fold changed after 5 weeks of chondrogenic differentiation as compared to undifferentiated hfMSC (week 0) using STRING9.0. Different clusters of interacting proteins can be identified, clusters A–F. Many other genes are identified that have no known protein interactions with other genes in the list.

In order to identify protein-protein interactions between proteins encoded by genes shown in table S2 STRING 9.0 (http://string-db.org) was used (figure 5). The clusters identified include many proteins involved in skeletal development and regulation of ECM homeostasis (figure 5 A, E), such as ACAN, COL2A1, ADAMTS5, PTH1R and GDF5, transcription regulators (figure 5C), such as JUN and FOS, as well as a node containing TGFβ superfamily proteins (figure 5B), such as GREM1. In addition, WNT11 and FZD2 seem to play a role in the chondrogenic differentiation of hfMSCs. We propose that if proteins in a related network mode change simultaneously, this increases the likelihood that these proteins play an important role in the differentiation of the hfMSCs toward the chondrogenic lineage.

### 2.2 hfMSCs acquire a partial GP phenotype after 5 weeks of differentiation

**Figure 6:**
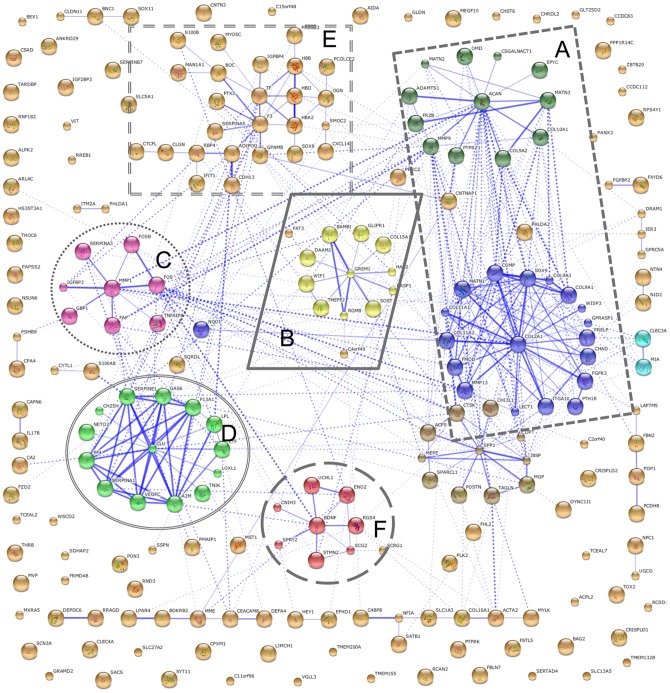
Protein interactions of differentially expressed genes between undifferentiated hfMSC and prepubertal growth plate cartilage. Analysis of protein interactions of genes that are differentially expressed in undifferentiated hfMSC (week 0) compared to average expression profiles of growth plate cartilage of 3 prepubertal donors using STRING 9.0. Large clusters of interacting proteins can be identified (A–F). Many other genes are identified that have no known protein interactions with other genes in the list.

Histological and gene expression analyses showed that the differentiating hfMSCs acquired a hyaline cartilage phenotype. In order to investigate how the expression profile changes from a MSC toward a GP profile we compared the gene profiles of the primary hfMSCs with prepubertal GP samples and identified many genes that are differentially expressed (table S3). In order to visualize the protein-protein interactions of the proteins encoded by these genes, we used STRING9.0. STRING (Search Tool for the retrieval of Interaction Genes/Proteins) is a freely available tool to visualize known and predicted protein interactions [12]. As such, it visualizes underlying protein networks that that are significantly changed within our data set. Interestingly, there is a large overlap of clusters identified in the profiles of MSC week 0 vs. MSC week 5 (figure 5) and the profile of MSC week 0 vs prepubertal GP (figure 6). Note that these clusters are based on protein-protein interactions, and not functional similarities. Protein clusters include ECM related proteins (figure 6A, E), TGFβ superfamily related proteins and their interactants (figure 6B), transcription regulatory proteins (figure 6C), and cytoskeleton related proteins (figure 6D. In addition, a large cluster containing proteins that are important in chondrocyte hypertrophy is visible in the MSC vs. GP data.

Figure 5A depicts a large cluster of ECM related proteins that change over 3.29 fold between week 0 vs. week 5. The expression of genes encoding cartilaginous ECM proteins, such as collagens (*COL1A1*, *COL1A2*, *COL2A1*, *COL5A2*, *COL11A1*), chondroadherin (*CHAD*), cartilage oligomeric matrix protein (*COMP*), secreted phosphoprotein 1 (osteopontin, *SPP1*), and fibronectin type III (*FNDC1*), was up regulated. Growth and differentiation factor 5 (GDF5), previously reported as stimulator of chondrocyte proliferation [13], was highly expressed at the earliest time point observed and down regulated thereafter.

Cluster 5B depicts proteins involved in TGFβ signaling. BMP2 and its downstream effector ID3 are up regulated early in differentiation consistent with previous reports on the importance of BMP signaling in chondrogenesis [14], [15]. The BMP inhibitor follistatin (FST) was highly expressed at the earliest time point, and down regulated at later times and was previously shown to be expressed by proliferative, but not by hypertrophic chondrocytes [16]. This is in concordance with the observation of Kearns et al, who described the activation of a feedback loop in which the expression of follistatin is induced by BMP2 [17].

Cluster 5C depicts proteins interactions with a variety of functions, such as regulation of transcription, coagulation, matrix mineralization and early hypertrophic differentiation. Members of the complement and coagulation family of proteins were expressed during *in vitro* chondrogenic differentiation. Coagulation factor XIII (*F13A1*) expression was up regulated, while other genes like complement component I (*C1R*), urokinase-type plasminogen activator (*PLAU*) and bradykinin receptor B2 (*BDKRB2*) were downregulated during differentiation in our model. These proteins are associated with matrix mineralization [18]–[20] or matrix degradation in the growth plate [21]–[23], indicating early hypertrophic differentiation in the model, but not late hypertrophy. Proto-oncogene KIT (cluster F, hypertrophy related genes), transcription factor JUN and FOS are all associated with the regulation of cell proliferation [24], [25]. Cluster 5C depicts several genes involved in the regulation of cell survival and proliferation and signal transduction. Caveolin 1 (*CAV1*), a multifunctional scaffolding protein located at cell surface caveolae, regulates TGF, Wnt, cytokine and VEGF signaling by modulating their downstream signaling cascades such as the JAK/STAT, β-catenin/LEF1, MAPK/ERK and PI3K/AKT signaling pathways [26]–[29]. In cluster D we find proteins involved in cell cycle regulation. Expression of the nuclear envelope protein lamin B2 (*LMNB2*, interacts with proteins in cluster D) was first up regulated and later in differentiation down regulated in our model. Constantinescu et al describe that B-type lamins are differentially expressed during different stages of ESC differentiation and suggest a role of these nuclear envelope proteins in the regulation of of the differentiation. [30]. The role for LMNB2 in chondrogenic differentiation is not known, but the changes in gene expression during chondrogenic differentiation may imply a role for regulation of the timing of hypertrophic differentiation in our in vitro cultures.

In figure 6 we identify the same clusters of interacting protein as in figure 5. The comparison between these images gives us insight into the route that MSCs take toward the differentiation into growth plate cartilage. The most obvious difference between the clusters identified in figure 5 and the ones in figure 6 is cluster 6E, which contains many proteins involved in late hypertrophic differentiation. Other genes showing significant differences in expression are clustered in the same groups, but the changes in gene expression profile between growth plate chondrocytes and undifferentiated MSCs is larger than that between week 0 and week 5 differentiated MSCs. The presence of the most important nodes in larger protein interaction clusters, emphasize that this cannot be a casualty. In the differentiating MSCs we identified a steep increase in expression of the early hypertrophic marker *COL10A1*, however, the addition of many more hypertrophic markers in the GP samples indicate that in order to fully form GP cartilage, MSCs will have to activate genes that induce later stages of hypertrophy.

## Discussion

### 3.1 hfMSCs can serve as a model for human growth plate development

In this study we identified many protein clusters involved in the differentiation of hfMSCs toward chondrocytes. In order for hfMSCs to serve as a model for the development of the epiphyseal growth plate, the cells need to obtain gene expression profiles that contain protein clusters that have been shown to play functional roles in the different stages of growth plate development. Fetal human bone marrow-derived MSCs seem to display better chondrogenic differentiation capacity than adult MSCs [10]. Consequently, it seems appropriate to use fetal bone marrow-derived MSCs as a model for chondrogenesis. However, fetal MSCs are not easily obtained, due to ethical and legal considerations, and as a consequence, adult bone marrow-derived MSCs have been used in most previous studies [31]–[34].

Previously, we have demonstrated that the hfMSCs differentiating towards chondrocytes acquired a GP cartilage-like phenotype [11]. Chondrogenic differentiation occurred in our *in vitro* model, as illustrated by the progressive increase in expression of the chondrocyte markers collagen type II and X and the cartilaginous matrix constituent glycosaminoglycan over time. Interestingly, hypertrophic differentiation as evidenced by an increase in cell size and positive staining for collagen X was only observed in a discrete zone of the pellet, which was surrounded at both sites by chondrocytes positive for collagen type II. Further confirmation of chondrogenesis was obtained by analysis of mRNA expression of cartilage markers, which, in addition, also validated our microarray results. Similar gene expression patterns were obtained during chondrogenic differentiation of fetal MSCs derived from other donors. This suggested that the selected 22 weeks old fetal MSC-donor was representative for fetal bone marrow in general.

Most studies in which MSCs are used to produce cartilage are aimed at obtaining articular cartilage. Cartilage matrix analysis is often limited to examining collagen type 2 and aggrecan expression. These markers are characteristic for hyaline cartilage and cannot distinguish growth plate cartilage from articular cartilage. Indeed, Huang et al. performed global microarray analysis of adult bovine MSCs at time 0 and after 28 days of differentiation in agarose constructs and compared the gene expression profile to that of chondrocytes isolated from articular cartilage. They showed that chondrogenically differentiating MSC do not form articular cartilage at 28 days in the presence of TGFβ3 [35]. This is in line with our present and previous study [11].

Although many pathways are involved in chondrocyte differentiation, all the studies leading to the identification of these pathways have been performed in animal chondrocytes and the number of studies using human chondrocytes is limited. A recent publication of our group identified 3 soluble antagonists to the Wnt and BMP pathway as highly expressed in articular cartilage and not growth plate cartilage [36]. In this article, we showed that when MSCs differentiate towards chondrocytes, they adopt a GP-like phenotype, whereas the addition of recombinant DKK1, GREM1 and FRZB during this differentiation results in inhibition of hypertrophic differentiation and mineralization. The data presented in the current manuscript shed light on the route taken for mesenchymal stromal cells toward the chondrogenic phenotype and may be useful in determining strategies for selective generation of either functional GP cartilage for research into growth plate (GP) maturation and fusion or generation of functional articular cartilage for tissue engineering strategies.

### 3.2 Differentiating hfMSCs show signs of early chondrogenesis, not endochondral ossification and matrix mineralization

In order to get insight into the requirement for a growth plate gene expression profile, we generated a map of the protein-protein interactions of the proteins of which the gene expression changed more than 3.29 fold. We compared that map to a map of protein-protein interactions of genes that change between undifferentiated MSCs and growth plate chondrocytes. We observed the same clusters of interacting proteins in both maps, and obtained clear evidence that there is early hypertrophic differentiation, as witnessed by the increased expression of collagen X. We obtained less evidence for late hypertrophic differentiation, although markers such as VEGF, LIF, PLAU, C1R and F13A1 are upregulated during week 4+5 of chondrogenic differentiation. We have found no indication of mineralization in our cultures. This indicates that the chondrogenic differentiation of hfMSCs is a good model for the early phases of growth plate development, but under these conditions, not for the late phase. Other reports, more specific in the field of bone tissue engineering have indicated necessary specific supplements, such as T3, dexamethasone and Wnt3a, for MSC differentiation into bone tissue, suggesting that when these factors are added to our cultures during the later stages of differentiation, one may observe induction of late hypertrophic markers and mineralization [37]–[39].

The model we describe offers insight into the route that MSCs take during the chondrogenic differentiated into growth plate cartilage as depicted in figure 6. This enables us to perform different types of research in the field of bone tissue engineering and early growth plate differentiation To our knowledge this is the first time human fetal mesenchymal stromal cells are followed on their route toward the chondrogenic differentiation. We followed the differentiation during 5 weeks with microarray data every week and compared the obtained gene profiles to the gene expression profile GP chondrocytes from prepubertal and fetal growth plate.

Using analysis of protein-protein interaction networks, we observed that the typical markers for chondrogenesis and early stages of hypertrophic differentiation are present, but other protein clusters, such as genes important for late hypertrophy and endochondral ossification are not induced. Using traditional analysis methods, these observations were likely to be missed, resulting in sub-optimal control of tissue formation. Moreover, the data provide insight into the use of human fetal mesenchymal stromal cells as an alternative to animal models for processes that are not regulated in the same manner in various species. As a model, it is readily accessible for genetic manipulation and might be used for unraveling the molecular mechanisms underlying growth regulation in the human epiphyseal plate during puberty. As such it may find its use in the development of novel treatment strategies for various growth disorders aimed at intervening in growth plate maturation and fusion and bone tissue engineering.

## Experimental Procedures

### 4.1 Cell culture

The use of human fetal material was approved by the medical ethical committee of the Leiden University Medical Center and written informed consent was obtained from the women undergoing elective abortion. Cell suspensions of fetal bone marrow were obtained by flushing the long bones of fetuses with M199 washing medium. For the chondrogenic differentiation and microarray analysis, cells derived from a single 22 week old fetus were used. Red cells were depleted by incubation for 10 minutes in NH_4_Cl (8.4 g/L)/KHCO_3_ (1 g/L) buffer at 4°C. Mononuclear cells were plated at a density of 16×10^4^ cells/cm^2^ in M199 culture medium (Gibco) supplemented with 10% fetal bovine serum (FBS), 1% penicillin/streptavidin (P/S), fungizone, endothelial cell growth factor (ECGF) 20 µg/ml (Roche Diagnostics) and heparin 8 U/ml in culture flasks coated with 1% gelatin according to previously established culture conditions for human fetal MSCs [40]. Cultures were kept in a humidified atmosphere at 37°C with 5% CO2. The culture medium was changed twice per week. After reaching near-confluence at passage 4 to 5 (15 population doublings), hfMSCs were harvested by treatment with 0.5% trypsin and 0.5% ethylenediaminetetraacetic acid (EDTA; Gibco) for 5 minutes at 37°C and replated for chondrogenic differentiation.

### 4.2 In vitro chondrogenic differentiation

hfMSCs (2×10^5^ cells/well) were cultured in cell pellets. Pellets were formed by centrifugation of the cells at 1200 rpm for 4 minutes in U-shaped 96-well suspension culture plates (Greiner). To induce chondrogenesis the pellets were cultured at 37°C with 5% CO_2_ in 200 µl of serum-free chondrogenic medium consisting of high-glucose (25 mM) Dulbecco's modified Eagle's medium (DMEM; Gibco) supplemented with 40 µg/ml proline (Sigma), 100 µg/ml sodium pyruvate (Sigma, USA), 50 mg/ml ITS (insulin-transferrin-selenic acid) with Premix (BD Biosciences), 1% Glutamax (Gibco), 1% penicillin/streptavidin, 50 µg/ml ascorbate-2-phosphate (Sigma), 10^−7^ M dexamethasone (Sigma), 10 ng/ml transforming growth factor-β3 (TGFβ3; R&D Systems), 500 ng/ml bone morphogenetic protein 6 (BMP6) and antibiotic and antimycotic mix (0.06% polymixin, 0.2% kanamycin, 0.2% penicillin, 0.2% streptavidin, 0.02% nystatin and 0.5% amphotericin) as described by Sekiya et al., 2001 [41]. The medium was changed twice per week for 5 weeks.

### 4.3 Histological analysis

Two pellets per time point (after 1, 2, 3, 4, or 5 weeks of chondrogenesis) were used for histological evaluation. Pellets were fixed in 10% formalin, dehydrated by treatment with graded ethanols and processed for paraffin embedding. 5 µm sections were cut using a Reichert Jung 2055 microtome (Leica). For each pellet, only the midsagittal sections were mounted on glass slides. Before histological (toluidine blue) or immunohistochemical staining, sections were deparaffinized in xylene, treated with graded ethanols followed by three washing steps with phosphate buffered saline (PBS).

For immunofluorescence of collagen type II, sections were pre-treated with 10 mM citric acid buffer (pH = 6) for antigen retrieval. Sections were incubated with a collagen type II monoclonal antibody (clone 3HH1-F9, Abnova) at 1∶100 dilution in 1% bovine serum albumine (BSA)/PBS buffer overnight at 4°C. After washing, sections were incubated with Alexa Fluor 488-Goat anti-Mouse IgG1 (Invitrogen, Molecular Probes, diluted 1∶1000 in PBS/1% BSA) for 1 hour and protected from light. Sections were counterstained with DAPI and mounted with vectashield.

For collagen type X immunohistochemistry, sections were preincubated with blocking buffer (1% H2O2 in 40% methanol, 60% tris buffered saline) twice for 15 minutes at room temperature, followed by overnight incubation at 4°C with mouse monoclonal antibody against collagen type X in a 1∶100 dilution (Quartett). Next, sections were incubated with the secondary antibody biotinylated rabbit-anti-mouse IgG (DAKO) in a 1∶300 dilution, followed by incubation with horseradish-peroxidase-conjugated-streptavidine (Amersham Biosciences). Staining was visualized with 3-amino-9-ethylcarbazole substrate in 0.2 mg/ml acetate buffer (pH 5.2) with 0.04% H_2_O_2_. After counterstaining with haematoxylin, the sections were mounted in Histomount (National Diagnostics). Pictures of the stained pellets were taken with a Nikon DXM 1200 digital camera using standardized settings.

### 4.4 Patients and tissue preparation

Human proximal tibia growth plate tissues were collected from patients at Tanner stage B1 undergoing surgery for different medical conditions. The protocol used was approved by the medical ethical committee of the Leiden University Medical Center and written informed consent was obtained from the patients as well as their parents. Epiphyseal samples were embedded in Tissue-Tek (Sakura Finetek Europ BV, Zoeterwoude, The Netherlands) and frozen in liquid nitrogen. RNA was extracted according to the optimized method for RNA extraction from cartilage as described by Heinrichs et al. [42].

### 4.5 RNA isolation from hfMSCs

Total RNA from 2×10^6^ undifferentiated hfMSCs derived from the 22 weeks old fetus was extracted with Trizol (Invitrogen). After 1, 2, 3, 4, or 5 weeks of chondrogenesis, 60 pellets (per time point) were pooled and homogenized in 1 ml 4 M guanidine isothiocyanate solution (Sigma) and RNA was extracted as described above. The extracted total RNA was purified using the RNeasy kit according to recommendations of the manufacturer (Qiagen).

### 4.6 Gene expression profiling

High RNA quality was confirmed by capillary electrophoresis on an Agilent 2100 bioanalyzer (Agilent). Total RNA (100 ng) was amplified and labeled using the GeneChip Two-Cycle cDNA Synthesis Kit (Affymetrix) and the MEGAscript T7 Kit (Ambion). For gene expression profiling, labeled cRNA was hybridized in duplicate to Affymetrix Human Genome U133 PLUS 2.0 Array Genechips. All procedures were carried out according to the manufacturer's recommendations.

Raw data from Affymetrix CEL files were analyzed using SAS software package Microarray Solution version 1.3 (SAS Institute). Custom CDF version 10 with Entrez based gene definitions [43] was applied to map the probes to genes. Gene annotation was obtained using the Affymetrix NetAffx website (http://www.affymetrix.com/analysis/index.affx). Quality control, normalization and statistical modeling were performed by array group correlation, mixed model normalization and mixed model analysis respectively. The normalized expression values for each gene were standardized by linearly scaling the values across all samples of the time course to a mean of 0 with an SD of 1. Analysis of differential gene expression was based on loglinear mixed model of perfect matches [44]. A false discovery rate of a = 0.05 with Bonferroni-correction for multiple testing was used to set the level of significance. The raw and normalized data are deposited in the Gene Expression Omnibus database (http://www.ncbi.nlm.nih.gov/geo/; accession no. GSE40942) and followed MIAME requirements.

### 4.7 Microarray data analysis

The statistical analysis of the microarray data was based on the normalized mean expression values per probe at 6 time points with 2 replications at each time point (12 observations per probe). In order to identify subgroups of probes with similar expression profiles over time, a principal component analysis (PCA) of the co-variance matrix was carried out on the mean expression value for each probe at each time point. For each probe, factor scores for principal components 1, 2 and 3 were obtained by regression analysis of the 12 array results (6 time points in duplicate) for that specific probe to those components. The first principal component corresponded with the general expression level during the whole experiment, whereas the second and third component corresponded with changes over time. Since our interest was to identify genes associated with the changes that occur during differentiation from stromal cells towards chondrocytes, we focused our analysis on the second and third component. By construction, these factor scores had a mean of 0 with an SD of 1. Generally, the distribution over the factor scores showed a normal distribution with outliers. We used a cut-off of ≥3.29 to select outlying probes. This cut-off would select 0.1% of the probes, if the factors scores would follow a pure normal distribution that could be expected if the data were pure noise. The presence of replications allowed us to assess the statistical significance of the factor scores and to remove probes that were not significant at the α = 5% level.

### 4.8 Pathway and network analysis

Using sets of probes emerging from PCA, a search for relevant KEGG pathways was performed using the DAVID® Knowledgebase, a publicly available bioinformatics tool for functional annotation (http://david.abcc.ncifcrf.gov). To investigate known and predicted associations between the protein products of the genes that were more than 3.29-fold changed between week 0 and week 5, we used STRING9.0 (string-db.org).

To visualize known and predicted associations between protein products of the genes that were differentially expressed between undifferentiated hfMSCs and pre-pubertal growth plate samples we used STRING9.0. Growth plates of 3 donor with Tanner stage B1 were included (one 12 year-old female with chondroid osteosarcoma, one 8 year-old female with osteosarcoma, one 10 year-old male with cerebral palsy).

### 4.9 Quantitative real-time polymerase chain reaction (qPCR)

RNA was transcribed into cDNA using the First Strand cDNA Synthesis kit for qPCR (Roche Diagnostics) according to the manufacturer's protocol. Specific primer sets (available at www.utwente.nl/tnw/DBE) were designed to amplify aggrecan (*ACAN*), pannexin 3 (*PANX3*), epiphycan (*EPYC*), collagen type II (*COL2A1*), and type X (*COL10A1*), SRY-box 9 (*SOX9*), WNT11, lymphoid enhancer-binding factor 1 (*LEF1*), Gremlin 1 (*GREM1*). β_2_-Microglobulin (*B2M*), and glyceraldehyde 3-phosphate dehydrogenase (*GAPDH*) were used as housekeeping genes. The expression of these housekeeping genes was stable during differentiation of hfMSCs as shown by the microarray analysis. In order to test donor inter-variation, differentiated MSCs isolated from other fetal donors were used for qPCR analysis as well.

All PCR reactions were performed in triplicate with 5 ng cDNA and according to the manufacturer's protocol of the iQ™ SYBR® Green Kit (Biorad) in a final volume of 20 µl. The cDNA was amplified using the following thermal cycling conditions: one cycle at 50°C for 2 min and 95°C for 10 min, followed by 40 cycles of 15 s at 95°C, 15 s at 60°C and 15 s at 72°C and finally a melting curve was generated using the mIQ Single-Color—Real-Time PCR System (BioRad Laboratories, Hercules, California, USA). Fluorescence spectra were recorded and the threshold cycle number (Ct) was calculated using the accompanying mIQ-software. For each time point mean Ct was calculated and from this value the fold difference in expression between undifferentiated hfMSCs and differentiating cells using the 2^−ΔΔCt^ method was calculated using *B2M* as a reference as described by Schmittgen and Livak, 2008 [45]. For visualization, this value was log-transformed.

## Supporting Information

Figure S1 **There is a good correlation between qPCR and microarray expression data.** For (A) *ACAN*, (B) *COL2A1*, (C) *COL10A1*, (D) *SOX9*, (E) *PANX3*, (F) *EPYC*, (G) *WNT11*, (H) *LEF1* and (I) *GREM1* during 5 weeks of chondrogenic differentiation of hfMSCs. qPCR data are expressed as delta delta CT values corrected for the housekeeping gene *B2M*. The primary y-axis (left) indicates the qPCR results as normalized mean fold expression on a log-scale. The secondary y-axis (right) indicates the microarray analysis results as least square means (lsm).
(TIF)

Table S1 **List of genes selected with principal component analysis.**
(PDF)

Table S2 **List of top hits of up regulated/down regulated genes at each time point with their fold change in expression compared to undifferentiated hfMSCs.**
(DOC)

Table S3 **List of top hits of differentially expressed genes of pre-pubertal GP with their fold change in expression compared to undifferentiated hfMSCs.**
(DOC)
