# Supplementary figures and images for: Fetal Mesenchymal Stromal Cells Differentiating towards Chondrocytes Acquire a Gene Expression Profile Resembling Human Growth Plate Cartilage
Source: PLoS One. 2012 Nov 5;7(11):e44561. doi: 10.1371/journal.pone.0044561 (PMC3489884; doi:10.1371/journal.pone.0044561)

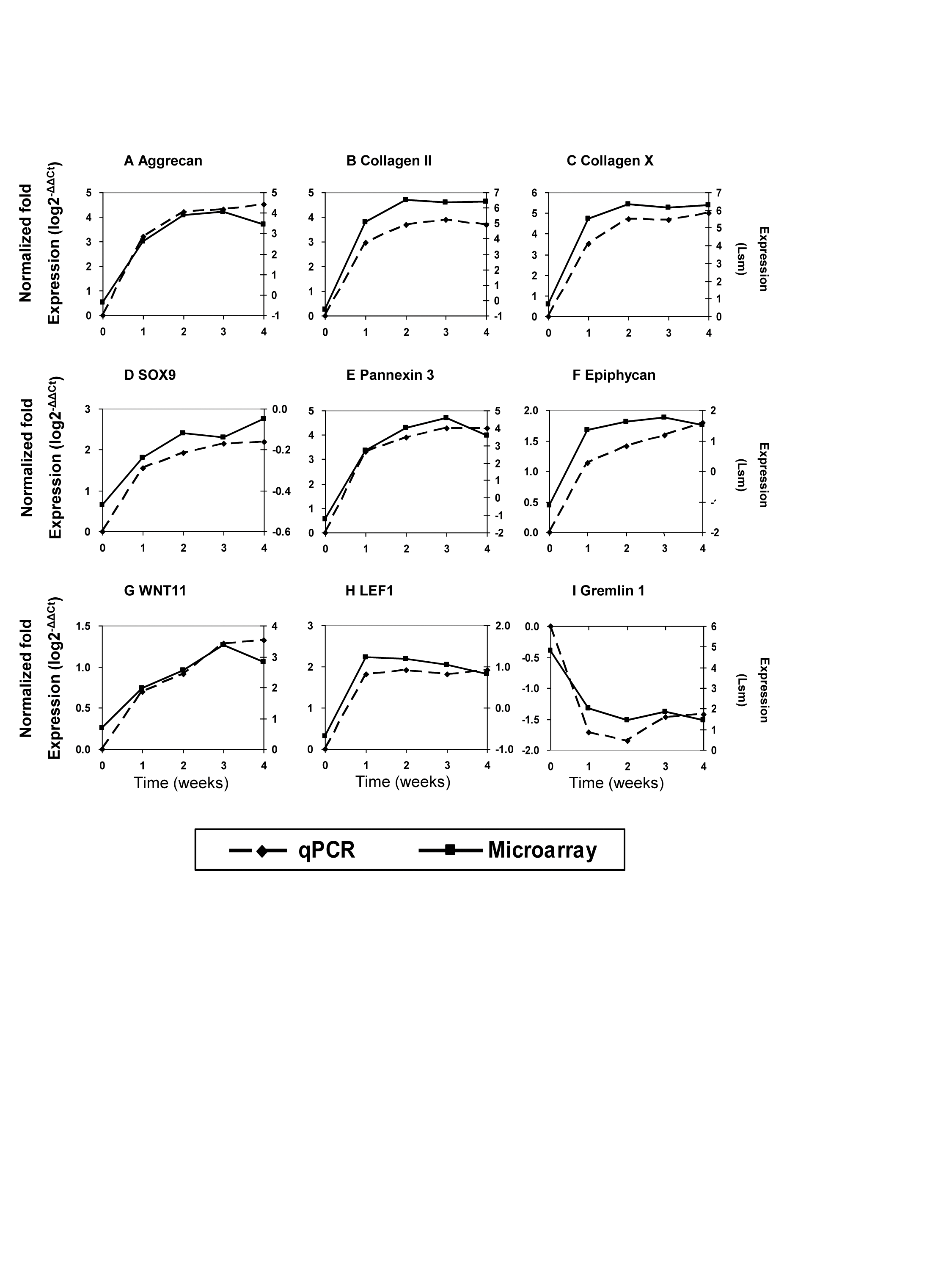

Supplement: Figure S1 — There is a good correlation between qPCR and microarray expression data. For (A) ACAN, (B) COL2A1, (C) COL10A1, (D) SOX9, (E) PANX3, (F) EPYC, (G) WNT11, (H) LEF1 and (I) GREM1 during 5 weeks of chondrogenic differentiation of hfMSCs. qPCR data are expressed as delta delta CT values corrected for the housekeeping gene B2M. The primary y-axis (left) indicates the qPCR results as normalized mean fold expression on a log-scale. The secondary y-axis (right) indicates the microarray analysis results as least square means (lsm). (TIF) [file pone.0044561.s001.tif]
