# Supplementary material for: Fetal Mesenchymal Stromal Cells Differentiating towards Chondrocytes Acquire a Gene Expression Profile Resembling Human Growth Plate Cartilage
Source: PLoS One. 2012 Nov 5;7(11):e44561. doi: 10.1371/journal.pone.0044561 (PMC3489884; doi:10.1371/journal.pone.0044561)
Supplement: Table S1 — List of genes selected with principal component analysis. (PDF) [file pone.0044561.s002.pdf]

Supplementary Table 1

Affymetrix ID	Gene code	Gene title
205856_at	SLC14A1	solute carrier family 14 (urea transporter), member 1 (Kidd blood group)
205911_at	PTHR1	parathyroid hormone receptor 1
219148_at	PBK	PDZ binding kinase
213182_x_at	CDKN1C	cyclin-dependent kinase inhibitor 1C (p57, Kip2)
206737_at	WNT11	wingless-type MMTV integration site family, member 11
203868_s_at	VCAM1	vascular cell adhesion molecule 1
218730_s_at	OGN	osteoglycin (osteoinductive factor, mimecan)
200665_s_at	SPARC	secreted protein, acidic, cysteine-rich (osteonectin)
223484_at	C15orf48	chromosome 15 open reading frame 48
206315_at	CRLF1	cytokine receptor-like factor 1
205497_at	ZNF175	zinc finger protein 175
204724_s_at	COL9A3	collagen, type IX, alpha 3
219410_at	TMEM45A	transmembrane protein 45A
218391_at	SNF8	SNF8, ESCRT-II complex subunit, homolog (S. cerevisiae)
210538_s_at	BIRC3	baculoviral IAP repeat-containing 3
201487_at	CTSC	cathepsin C
219134_at	ELTD1	EGF, latrophilin and seven transmembrane domain containing 1
212551_at	CAP2	CAP, adenylate cyclase-associated protein, 2 (yeast)
206421_s_at	SERPINB7	serpin peptidase inhibitor, clade B (ovalbumin), member 7
219837_s_at	CYTL1	cytokine-like 1
210220_at	FZD2	frizzled homolog 2 (Drosophila)
207064_s_at	AOC2	amine oxidase, copper containing 2 (retina-specific)
218542_at	CEP55	centrosomal protein 55kDa
206423_at	ANGPTL7	angiopoietin-like 7
231227_at	---	Transcribed locus, strongly similar to WNT-5A protein precursor
229494_s_at	CD63	CD63 molecule
223734_at	OSAP	ovary-specific acidic protein
206614_at	GDF5	growth differentiation factor 5 (cartilage-derived morphogenetic protein-1)
205713_s_at	COMP	cartilage oligomeric matrix protein
230372_at	---	Transcribed locus, PREDICTED: similar to hyaluronan synthase 2 [Pan troglodytes]
1563724_at	SACS	Spastic ataxia of Charlevoix-Saguenay (sacsin)
203499_at	EPHA2	EPH receptor A2
1556499_s_at	COL1A1	collagen, type I, alpha 1
219230_at	TMEM100	transmembrane protein 100
206790_s_at	NDUFB1	NADH dehydrogenase (ubiquinone) 1 beta subcomplex, 1, 7kDa
204825_at	MELK	maternal embryonic leucine zipper kinase
212565_at	STK38L	serine/threonine kinase 38 like
1554997_a_at	PTGS2	prostaglandin-endoperoxide synthase 2 (prostaglandin G/H synthase & cyclooxygenase)
204894_s_at	AOC3	amine oxidase, copper containing 3 (vascular adhesion protein 1)
203886_s_at	FBLN2	fibulin 2
203153_at	IFIT1	interferon-induced protein with tetratricopeptide repeats 1
242517_at	KISS1R	KISS1 receptor
1552340_at	SP7	Sp7 transcription factor
203963_at	CA12	carbonic anhydrase XII
1554950_at	AGC1	aggrecan 1 (chondroitin sulfate proteoglycan 1, large aggregating proteoglycan)
232451_at	---	MRNA; cDNA DKFZp564I0816 (from clone DKFZp564I0816)
227705_at	TCEAL7	transcription elongation factor A (SII)-like 7
1570574_at	GPR177	G protein-coupled receptor 177
218273_s_at	PPM2C	protein phosphatase 2C, magnesium-dependent, catalytic subunit
224735_at	CYBASC3	cytochrome b, ascorbate dependent 3
239787_at	KCTD4	potassium channel tetramerisation domain containing 4
226281_at	DNER	delta-notch-like EGF repeat-containing transmembrane
218839_at	HEY1	hairy/enhancer-of-split related with YRPW motif 1
214710_s_at	CCNB1	cyclin B1
231798_at	NOG	Noggin
204595_s_at	STC1	stanniocalcin 1
209189_at	FOS	v-fos FBJ murine osteosarcoma viral oncogene homolog
203297_s_at	JARID2	Jumonji, AT rich interactive domain 2
230137_at	TMEM155	transmembrane protein 155

208078_s_at	SNF1LK	SNF1-like kinase /// SNF1-like kinase
217989_at	DHRS8	dehydrogenase/reductase (SDR family) member 8
229125_at	ANKRD38	ankyrin repeat domain 38
205141_at	ANG /// RNASE4	angiogenin, ribonuclease, RNase A family, 5 /// ribonuclease, RNase A family, 4
204712_at	WIF1	WNT inhibitory factor 1
1552960_at	LRRC15	leucine rich repeat containing 15
225155_at	SNHG5	small nucleolar RNA host gene (non-protein coding) 5
204351_at	S100P	S100 calcium binding protein P
1569372_at	TUBB2B	Tubulin, beta 2B
205097_at	SLC26A2	solute carrier family 26 (sulfate transporter), member 2
204881_s_at	UGCG	UDP-glucose ceramide glucosyltransferase
203434_s_at	MME	membrane metallo-endopeptidase (neutral endopeptidase, enkephalinase)
1568574_x_at	SPP1	Secreted phosphoprotein 1 (osteopontin, bone sialoprotein I)
206908_s_at	CLDN11	claudin 11 (oligodendrocyte transmembrane protein)
1556153_s_at	NFKBIZ	Nuclear factor of kappa light polypeptide gene enhancer in B-cells inhibitor, zeta
210643_at	TNFSF11	tumor necrosis factor (ligand) superfamily, member 11, RANKL
203305_at	F13A1	coagulation factor XIII, A1 polypeptide
213791_at	PENK	proenkephalin
242324_x_at	CCBE1	collagen and calcium binding EGF domains 1
213338_at	TMEM158	transmembrane protein 158
213139_at	SNAI2	snail homolog 2 (Drosophila)
217979_at	TSPAN13	Tetraspanin 13
215420_at	IHH	Indian hedgehog homolog (Drosophila)
229645_at	C18orf51	chromosome 18 open reading frame 51
218717_s_at	LEPREL1	leprecan-like 1
238332_at	ANKRD29	ankyrin repeat domain 29
205828_at	MMP3	matrix metallopeptidase 3 (stromelysin 1, progelatinase)
209395_at	CHI3L1	chitinase 3-like 1 (cartilage glycoprotein-39)
204337_at	RGS4	regulator of G-protein signalling 4
201939_at	PLK2	polo-like kinase 2 (Drosophila)
228844_at	SLC13A5	solute carrier family 13 (sodium-dependent citrate transporter), member 5
218468_s_at	GREM1	gremlin 1, cysteine knot superfamily, homolog (Xenopus laevis)
201467_s_at	NQO1	NAD(P)H dehydrogenase, quinone 1
224482_s_at	RAB11FIP4	RAB11 family interacting protein 4 (class II)
206239_s_at	SPINK1	serine peptidase inhibitor, Kazal type 1
213492_at	COL2A1	collagen, type II, alpha 1
1552737_s_at	WWP2	WW domain containing E3 ubiquitin protein ligase 2
204162_at	KNTC2	kinetochore associated 2
213622_at	COL9A2	collagen, type IX, alpha 2
202497_x_at	SLC2A3	solute carrier family 2 (facilitated glucose transporter), member 3
206309_at	LECT1	leukocyte cell derived chemotaxin 1
1556427_s_at	LOC221091	similar to hypothetical protein
201762_s_at	PSME2	proteasome (prosome, macropain) activator subunit 2 (PA28 beta)
201795_at	LBR	lamin B receptor
209946_at	VEGFC	vascular endothelial growth factor C
210432_s_at	SCN3A	sodium channel, voltage-gated, type III, alpha
206439_at	DSPG3	dermatan sulfate proteoglycan 3
203498_at	DSCR1L1	Down syndrome critical region gene 1-like 1
202912_at	ADM	adrenomedullin
221729_at	COL5A2	collagen, type V, alpha 2
1555345_at	SLC38A4	solute carrier family 38, member 4
210095_s_at	IGFBP3	insulin-like growth factor binding protein 3
201601_x_at	IFITM1	interferon induced transmembrane protein 1 (9-27)
205483_s_at	ISG15	ISG15 ubiquitin-like modifier
1554685_a_at	KIAA1199	KIAA1199
221019_s_at	COLEC12	collectin sub-family member 12 /// collectin sub-family member 12
240448_at	KIAA0802	KIAA0802
200790_at	ODC1	ornithine decarboxylase 1
206932_at	CH25H	cholesterol 25-hydroxylase
205352_at	SERPINI1	serpin peptidase inhibitor, clade I (neuroserpin), member 1
228640_at	---	CDNA clone IMAGE:4800096
205051_s_at	KIT	v-kit Hardy-Zuckerman 4 feline sarcoma viral oncogene homolog
204731_at	TGFBR3	transforming growth factor, beta receptor III (betaglycan, 300kDa)

221823_at	C5orf30	chromosome 5 open reading frame 30
1554736_at	ARHGAP29	Rho GTPase activating protein 29
217997_at	PHLDA1	pleckstrin homology-like domain, family A, member 1
226907_at	PPP1R14C	protein phosphatase 1, regulatory (inhibitor) subunit 14C
223235_s_at	SMOC2	SPARC related modular calcium binding 2
202403_s_at	COL1A2	collagen, type I, alpha 2
204469_at	PTPRZ1	protein tyrosine phosphatase, receptor-type, Z polypeptide 1
223614_at	C8orf57	chromosome 8 open reading frame 57
212850_s_at	LRP4	low density lipoprotein receptor-related protein 4
202965_s_at	CAPN6	calpain 6
223316_at	CCDC3	coiled-coil domain containing 3
200974_at	ACTA2	actin, alpha 2, smooth muscle, aorta
213293_s_at	TRIM22	tripartite motif-containing 22
222020_s_at	HNT	neurotrimin
210609_s_at	TP53I3	tumor protein p53 inducible protein 3
201739_at	SGK	serum/glucocorticoid regulated kinase
217995_at	SQRDL	sulfide quinone reductase-like (yeast)
204682_at	LTBP2	latent transforming growth factor beta binding protein 2
201195_s_at	SLC7A5	solute carrier family 7 (cationic amino acid transporter, y+ system), member 5
206764_x_at	MPPE1	metallophosphoesterase 1
213060_s_at	CHI3L2	chitinase 3-like 2 /// chitinase 3-like 2
205334_at	S100A1	S100 calcium binding protein A1
209955_s_at	FAP	fibroblast activation protein, alpha
204035_at	SCG2	secretogranin II (chromogranin C)
217875_s_at	TMEPAI	transmembrane, prostate androgen induced RNA
203879_at	PIK3CD	phosphoinositide-3-kinase, catalytic, delta polypeptide
202709_at	FMOD	fibromodulin
1554737_at	FBN2	fibrillin 2 (congenital contractural arachnodactyly)
205941_s_at	COL10A1	collagen, type X, alpha 1(Schmid metaphyseal chondrodysplasia)
202727_s_at	IFNGR1	interferon gamma receptor 1
226930_at	FNDC1	fibronectin type III domain containing 1
207001_x_at	TSC22D3	TSC22 domain family, member 3
206960_at	GPR23	G protein-coupled receptor 23
203666_at	CXCL12	chemokine (C-X-C motif) ligand 12 (stromal cell-derived factor 1)
204320_at	COL11A1	collagen, type XI, alpha 1
203058_s_at	PAPSS2	3'-phosphoadenosine 5'-phosphosulfate synthase 2
205870_at	BDKRB2	bradykinin receptor B2
201464_x_at	JUN	v-jun sarcoma virus 17 oncogene homolog (avian)
226989_at	RGMB	RGM domain family, member B
229740_at	LOC643008	PP12104
203304_at	BAMBI	BMP and activin membrane-bound inhibitor homolog (Xenopus laevis)
218899_s_at	BAALC	brain and acute leukemia, cytoplasmic
224348_s_at	H19	H19, imprinted maternally expressed untranslated mRNA
209560_s_at	DLK1	delta-like 1 homolog (Drosophila)
222162_s_at	ADAMTS1	ADAM metallopeptidase with thrombospondin type 1 motif, 1
206115_at	EGR3	early growth response 3
1562094_at	MGC26963	Hypothetical protein MGC26963
216952_s_at	LMNB2	lamin B2
210948_s_at	LEF1	lymphoid enhancer-binding factor 1
1563466_at	MYLK	Myosin, light polypeptide kinase
212689_s_at	JMJD1A	jumonji domain containing 1A
205347_s_at	TMSL8	thymosin-like 8
204967_at	SHROOM2	shroom family member 2
218009_s_at	PRC1	protein regulator of cytokinesis 1
212067_s_at	C1R /// LOC643676	complement component 1, r subcomponent
1560259_at	RORA	RAR-related orphan receptor A
206432_at	HAS2	hyaluronan synthase 2
1561065_at	ANKRD6	Ankyrin repeat domain 6
1555800_at	ZNF533	zinc finger protein 533
219747_at	C4orf31	chromosome 4 open reading frame 31
1558636_s_at	ADAMTS5	ADAM metallopeptidase with thrombospondin type 1 motif, 5 (aggrecanase-2)
227497_at	---	CDNA FLJ11723 fis, clone HEMBA1005314
1555527_at	COL9A1	collagen, type IX, alpha 1

202768_at	FOSB	FBJ murine osteosarcoma viral oncogene homolog B
204221_x_at	GLIPR1	GLI pathogenesis-related 1 (glioma)
204774_at	EVI2A	ecotropic viral integration site 2A
206157_at	PTX3	pentraxin-related gene, rapidly induced by IL-1 beta
202643_s_at	TNFAIP3	tumor necrosis factor, alpha-induced protein 3
234994_at	KIAA1913	KIAA1913
227475_at	FOXQ1	forkhead box Q1
219334_s_at	OBFC2A	oligonucleotide/oligosaccharide-binding fold containing 2A
218986_s_at	FLJ20035	hypothetical protein FLJ20035
228382_at	FAM105B	family with sequence similarity 105, member B
205523_at	HAPLN1	hyaluronan and proteoglycan link protein 1
224967_at	UGCG	UDP-glucose ceramide glucosyltransferase
213817_at	---	CDNA FLJ13601 fis, clone PLACE1010069
212900_at	SEC24A	SEC24 related gene family, member A (S. cerevisiae)
1552619_a_at	ANLN	anillin, actin binding protein
224609_at	SLC44A2	solute carrier family 44, member 2
203755_at	BUB1B	BUB1 budding uninhibited by benzimidazoles 1 homolog beta (yeast)
1555724_s_at	TAGLN	transgelin
202450_s_at	CTSK	cathepsin K (pycnodysostosis)
213861_s_at	FAM119B	family with sequence similarity 119, member B
213248_at	LOC221362	hypothetical protein LOC221362
203570_at	LOXL1	lysyl oxidase-like 1
230407_at	---	Transcribed locus, strongly similar to strawberry notch homolog 1; MOP-3
209567_at	RRS1	RRS1 ribosome biogenesis regulator homolog (S. cerevisiae)
210512_s_at	VEGF	vascular endothelial growth factor
205289_at	BMP2	bone morphogenetic protein 2
203065_s_at	CAV1	caveolin 1, caveolae protein, 22kDa
203758_at	CTSO	cathepsin O
205476_at	CCL20	chemokine (C-C motif) ligand 20
207826_s_at	ID3	inhibitor of DNA binding 3, dominant negative helix-loop-helix protein
205479_s_at	PLAU	plasminogen activator, urokinase
201136_at	PLP2	proteolipid protein 2 (colonic epithelium-enriched)
203764_at	DLG7	discs, large homolog 7 (Drosophila)
209160_at	AKR1C3	aldo-keto reductase family 1, member C3 (3-alpha hydroxysteroid dehydrogenase, type II
207977_s_at	DPT	dermatopontin
205125_at	PLCD1	phospholipase C, delta 1
207980_s_at	CITED2	Cbp/p300-interacting transactivator, with Glu/Asp-rich carboxy-terminal domain, 2
204475_at	MMP1	matrix metallopeptidase 1 (interstitial collagenase)
1556209_at	CLEC2B	C-type lectin domain family 2, member B
205830_at	CLGN	calmegin
219295_s_at	PCOLCE2	procollagen C-endopeptidase enhancer 2
205907_s_at	OMD	osteomodulin
206869_at	CHAD	chondroadherin
223836_at	KSP37	Ksp37 protein
204948_s_at	FST	follistatin
240955_at	PANX3	pannexin 3

)
