# Supplementary material for: Fetal Mesenchymal Stromal Cells Differentiating towards Chondrocytes Acquire a Gene Expression Profile Resembling Human Growth Plate Cartilage
Source: PLoS One. 2012 Nov 5;7(11):e44561. doi: 10.1371/journal.pone.0044561 (PMC3489884; doi:10.1371/journal.pone.0044561)
Supplement: Table S2 — List of top hits of up regulated/down regulated genes at each time point with their fold change in expression compared to undifferentiated hfMSCs. (DOC) [file pone.0044561.s003.doc]

Table S2. List of genes that are ≥3.29-fold changed from week 0 to week 5. Fold- changes between weeks 1-5 as compared to week 0 are listed.

| **DESCRIPTION** | **GENE_SYM** | **1 week** | **2 week** | **3 week** | **4 week** | **5 week** |
| --- | --- | --- | --- | --- | --- | --- |
| Collagen, type II, alpha 1 | COL2A1 | 48,7 | 127,5 | 124,6 | 86,8 | 118,4 |
| Collagen, type IX, alpha 3 | COL9A3 | 17,4 | 56,7 | 53,0 | 31,3 | 64,2 |
| Cartilage oligomeric matrix protein | COMP | 29,5 | 63,4 | 55,0 | 16,6 | 49,0 |
| S100 calcium binding protein P | S100P | 23,6 | 39,4 | 47,6 | 25,2 | 45,3 |
| Collagen, type X, alpha 1 | COL10A1 | 26,3 | 43,1 | 42,8 | 43,5 | 43,2 |
| secreted phosphoprotein 1 | SPP1 | 29,2 | 38,1 | 29,1 | 30,3 | 36,7 |
| Pannexin 3 | PANX3 | 14,1 | 35,2 | 56,7 | 17,3 | 36,6 |
| H19, imprinted maternally expressed untranslated mRNA | H19 | 20,7 | 37,5 | 37,8 | 13,7 | 27,3 |
| Angiopoietin-like 7 | ANGPTL7 | 7,2 | 20,8 | 24,4 | 14,4 | 24,1 |
| osteomodulin | OMD | 14,8 | 26,7 | 28,9 | 10,2 | 22,8 |
| Fibromodulin | FMOD | 4,8 | 16,4 | 19,8 | 10,9 | 22,1 |
| aggregan | ACAN | 7,7 | 17,5 | 21,6 | 9,2 | 19,1 |
| SPARC related modular calcium binding 2 | SMOC2 | 4,9 | 10,4 | 18,2 | 7,8 | 16,6 |
| solute carrier family 13 (sodium-dependent citrate transporter), member 5 | SLC13A5 | 3,8 | 10,9 | 16,6 | 7,1 | 13,5 |
| parathyroid hormone 1 receptor | PTHR1 | 3,2 | 8,7 | 9,9 | 4,5 | 11,4 |
| Fibroblast growth factor binding protein 2 | KSP37 | 4,4 | 11,8 | 11,1 | 5,0 | 11,1 |
| procollagen C-endopeptidase enhancer 2 | PCOLCE2 | 3,7 | 7,5 | 10,4 | 6,1 | 10,6 |
| Calpain 6 | CAPN6 | 2,5 | 5,9 | 9,9 | 8,1 | 9,8 |
| Sp7 transcription factor | SP7 | 11,0 | 12,0 | 14,7 | 4,1 | 9,6 |
| Fibronectin type III domain containing 1 | FNDC1 | 7,8 | 14,9 | 12,1 | 5,1 | 9,4 |
| Cytokine-like 1 | CYTL1 | 1,0 | 2,1 | 3,4 | 6,4 | 9,3 |
| BMP and activin membrane-bound inhibitor homolog | BAMBI | 3,3 | 5,9 | 8,4 | 4,2 | 8,8 |
| Optineurin | CCDC3 | 2,5 | 7,5 | 8,4 | 5,0 | 8,8 |
| Ankyrin repeat domain 38 | ANKRD38 | 21,0 | 15,0 | 14,3 | 4,1 | 8,3 |
| Amine oxidase, copper containing 2 | AOC2 | 4,6 | 5,7 | 7,5 | 3,6 | 8,3 |
| Epiphycan | EPYC | 5,2 | 6,6 | 7,7 | 4,3 | 8,0 |
| Serpin peptidase inhibitor, clade A (alpha-1 antiproteinase, antitrypsin), member 3 | SERPINA3 | 4,5 | 6,7 | 6,2 | 3,7 | 7,5 |
| G protein-coupled receptor 23 | GPR23 | 3,2 | 7,8 | 8,2 | 5,6 | 7,0 |
| Protein tyrosine phosphatase, receptor-type, Z polypeptide 1 | PTPRZ1 | 2,6 | 4,4 | 7,8 | 5,0 | 6,9 |
| 3'-phosphoadenosine 5'-phosphosulfate synthase 2 | PAPSS2 | 3,2 | 5,1 | 8,6 | 4,8 | 6,5 |
| Chondroadherin | CHAD | 2,1 | 4,6 | 5,4 | 4,7 | 6,3 |
| collagen, type XXIV, alpha 1 | COL24A1 | 2,7 | 4,3 | 5,7 | 3,5 | 6,0 |
| Serine/threonine kinase 38 like | STK38L | 2,3 | 5,5 | 7,5 | 3,5 | 6,0 |
| Solute carrier family 7 (cationic amino acid transporter, y+ system), member 5 | SLC7A5 | 4,0 | 5,4 | 8,7 | 2,9 | 6,0 |
| tetraspanin 13 | TSPAN13 | 2,0 | 3,1 | 5,0 | 4,5 | 5,6 |
| Amine oxidase, copper containing 3 (vascular adhesion protein 1) | AOC3 | 4,5 | 4,9 | 5,8 | 2,7 | 5,5 |
| osteoglycin | OGN | 7,4 | 9,4 | 10,5 | 3,1 | 5,4 |
| SRY (sex determining region Y)-box 8 | SOX8 | 2,4 | 4,6 | 6,6 | 2,6 | 5,2 |
| Solute carrier family 26 (sulfate transporter), member 2 | SLC26A2 | 1,5 | 3,3 | 3,8 | 2,9 | 5,2 |
| Tubulin, beta 2B | TUBB-PARALOG | 2,2 | 3,2 | 5,1 | 4,1 | 5,1 |
| Wingless-type MMTV integration site family, member 11 | WNT11 | 2,3 | 3,1 | 6,0 | 3,1 | 5,1 |
| Leukocyte cell derived chemotaxin 1 | LECT1 | 1,3 | 3,1 | 4,9 | 3,0 | 5,1 |
| RecQ protein-like 5 | MYO15B | 2,9 | 4,1 | 7,0 | 3,6 | 5,0 |
| Collagen, type XI, alpha 1 | COL11A1 | 3,9 | 5,5 | 5,6 | 4,2 | 4,9 |
| Dermatopontin | DPT | 3,1 | 4,9 | 5,7 | 3,2 | 4,8 |
| Coagulation factor XIII, A1 polypeptide | F13A1 | 1,7 | 3,5 | 3,4 | 4,3 | 4,6 |
| leucine rich repeat containing 1 | LRRC1 | 1,8 | 3,7 | 4,8 | 2,2 | 4,5 |
| Solute carrier family 38, member 4 | SLC38A4 | 1,5 | 2,3 | 3,0 | 4,3 | 4,5 |
| Proenkephalin | PENK | 3,6 | 3,5 | 4,2 | 3,6 | 4,3 |
| transmembrane protein 100 | FLJ10970 | 1,9 | 2,0 | 4,9 | 3,1 | 4,2 |
| Protein phosphatase 1, regulatory (inhibitor) subunit 14C | PPP1R14C | 4,5 | 3,7 | 5,0 | 2,2 | 4,2 |
| collagen, type IX, alpha 2 | COL9A2 | 2,0 | 3,3 | 3,8 | 2,5 | 4,1 |
| inhibitor of DNA binding 3, dominant negative helix-loop-helix protein | ID3 | 2,6 | 3,1 | 4,0 | 2,1 | 4,0 |
| Leprecan-like 1 | LEPREL1 | 1,2 | 1,6 | 4,7 | 2,5 | 4,0 |
| cyclin-dependent kinase inhibitor 1C (p57, Kip2) | CDKN1C | 1,2 | 2,0 | 3,8 | 3,4 | 3,9 |
| WNT inhibitory factor 1 | WIF1 | 7,9 | 6,5 | 4,2 | 2,8 | 3,6 |
| carbonic anhydrase XII | CA12 | 2,0 | 3,3 | 4,4 | 2,1 | 3,6 |
| low density lipoprotein receptor-related protein 4 | LRP4 | 4,2 | 3,3 | 3,2 | 2,4 | 3,6 |
| retinol binding protein 4, plasma | RBP4 | 2,0 | 3,9 | 4,3 | 1,9 | 3,5 |
| S100 calcium binding protein A1 | S100A1 | 2,7 | 3,3 | 3,2 | 3,2 | 3,5 |
| cytokine receptor-like factor 1 | CRLF1 | 3,5 | 3,8 | 4,0 | 2,2 | 3,4 |
| chemokine (C-X-C motif) ligand 13 | CXCL13 | 1,6 | 2,1 | 2,7 | 3,1 | 3,4 |
| hairy/enhancer-of-split related with YRPW motif 1 | HEY1 | 4,0 | 3,1 | 4,1 | 2,5 | 3,4 |
| prostate transmembrane protein, androgen induced 1 | TMEPAI | 3,4 | 4,1 | 3,6 | 1,7 | 3,3 |
| Rho GTPase activating protein 29 | ARHGAP29 | -2,6 | -2,9 | -3,5 | -3,2 | -3,3 |
| v-kit Hardy-Zuckerman 4 feline sarcoma viral oncogene homolog | KIT | -3,0 | -3,3 | -3,8 | -2,7 | -3,3 |
| Niemann-Pick disease, type C2 | NPC2 | -2,1 | -3,0 | -3,3 | -4,7 | -3,3 |
| brain abundant, membrane attached signal protein 1 | BASP1 | -1,8 | -2,6 | -2,9 | -5,7 | -3,3 |
|  | SART2 | -1,9 | -2,9 | -2,9 | -5,1 | -3,3 |
| protein phosphatase 4, regulatory subunit 1 | PPP4R1 | -3,1 | -4,0 | -2,7 | -4,5 | -3,4 |
| anillin, actin binding protein | ANLN | -2,3 | -2,9 | -2,9 | -2,7 | -3,4 |
| transmembrane protein 155 | FLJ30834 | -3,3 | -3,5 | -3,6 | -2,8 | -3,4 |
| adenylate kinase 3 | AK3 | -2,2 | -3,6 | -3,0 | -4,8 | -3,4 |
|  | IGSF4 | -2,2 | -2,4 | -3,2 | -3,5 | -3,4 |
| bradykinin receptor B2 | BDKRB2 | -2,9 | -3,3 | -3,5 | -3,1 | -3,4 |
| EGF, latrophilin and seven transmembrane domain containing 1 | ELTD1 | -2,3 | -2,7 | -3,2 | -3,8 | -3,4 |
| Beta-1,3-N-acetylglucosaminyltransferase 6 | B3GNT6 | -2,3 | -2,9 | -3,0 | -4,0 | -3,4 |
| sodium channel, voltage-gated, type III, alpha subunit | SCN3A | -2,9 | -3,1 | -3,6 | -2,8 | -3,4 |
| ubiquitin-conjugating enzyme E2C | UBE2C | -2,0 | -3,2 | -2,8 | -3,9 | -3,4 |
| origin recognition complex, subunit 6 like (yeast) | ORC6L | -1,7 | -2,7 | -2,7 | -3,4 | -3,4 |
| tubulin, **alpha** 1b | K-ALPHA-1 | -2,9 | -5,7 | -2,5 | -3,7 | -3,4 |
| lamin B2 | LMNB2 | -3,0 | -3,8 | -3,5 | -4,0 | -3,5 |
| protocadherin 7 | PCDH7 | -3,3 | -3,4 | -3,8 | -3,2 | -3,5 |
| calmegin | CLGN | -2,6 | -4,4 | -4,1 | -6,1 | -3,5 |
| cathepsin B | CTSB | -2,0 | -2,9 | -3,2 | -4,4 | -3,5 |
| cyclin G1 | CCNG1 | -2,6 | -3,8 | -4,2 | -6,9 | -3,6 |
| follistatin-like 5 | FSTL5 | -3,2 | -3,4 | -3,7 | -3,0 | -3,6 |
| cathepsin C | CTSC | -2,9 | -2,7 | -3,6 | -3,7 | -3,6 |
| NAD(P)H dehydrogenase, quinone 1 | NQO1 | -4,1 | -4,2 | -4,0 | -4,0 | -3,6 |
| discs, large (Drosophila) homolog-associated protein | DLG7 | -2,4 | -3,2 | -3,1 | -3,2 | -3,6 |
| Ovary-specific acidic protein | OSAP | -2,7 | -2,9 | -3,2 | -3,6 | -3,7 |
| follistatin | FST | -3,0 | -3,4 | -3,9 | -3,3 | -3,7 |
| brain and acute leukemia, cytoplasmic | BAALC | -3,5 | -3,2 | -3,8 | -3,7 | -3,7 |
| cathepsin O | CTSO | -3,4 | -3,6 | -3,2 | -4,1 | -3,8 |
| ARP3 actin-related protein 3 homolog (yeast) | ACTR3 | -2,5 | -3,8 | -2,7 | -6,3 | -3,8 |
| heat shock 70kDa protein 8 | HSPA8 | -3,1 | -4,6 | -4,1 | -8,4 | -3,8 |
| aldo-keto reductase family 1, member C3 (3-alpha hydroxysteroid dehydrogenase,  type II | AKR1C3 | -3,4 | -3,6 | -4,5 | -3,2 | -3,8 |
|  | LOC391047 | -3,2 | -3,7 | -3,4 | -4,2 | -3,9 |
| ISG15 ubiquitin-like modifie | G1P2 | -3,4 | -3,7 | -4,3 | -3,7 | -3,9 |
| pyruvate dehyrogenase phosphatase catalytic subunit 1 | PPM2C | -3,5 | -3,5 | -4,2 | -4,4 | -3,9 |
| serum/glucocorticoid regulated kinase | SGK | -2,0 | -4,1 | -4,8 | -6,1 | -3,9 |
| Collagen and calcium binding EGF domains 1 | CCBE1 | -3,1 | -3,5 | -4,9 | -4,0 | -3,9 |
| Sulfide quinone reductase-like (yeast) | SQRDL | -2,2 | -4,1 | -3,8 | -5,0 | -4,0 |
| NDC80 homolog, kinetochore complex component | KNTC2 | -3,0 | -3,5 | -3,5 | -4,1 | -4,0 |
| Complement component 1, r subcomponent-like | C1RL | -2,2 | -3,1 | -3,7 | -4,2 | -4,0 |
| ADP-ribosylation factor-like 6 interacting protein 1 | ARL6IP | -1,4 | -2,6 | -3,6 | -7,2 | -4,1 |
| spastic ataxia of Charlevoix-Saguenay (**sacs**in) | SACS | -2,8 | -3,3 | -3,2 | -6,4 | -4,2 |
| KIAA1199 | KIAA1199 | -3,2 | -4,5 | -4,5 | -3,9 | -4,3 |
| Latent transforming growth factor beta binding protein 2 | LTBP2 | -3,5 | -4,1 | -4,0 | -4,2 | -4,3 |
| Vascular endothelial growth factor C | VEGFC | -2,3 | -3,1 | -4,7 | -5,1 | -4,3 |
| fragile site, aphidicolin type, common, fra(7)(p22) | FRA7B | -3,4 | -3,6 | -4,1 | -5,0 | -4,3 |
| hydroxysteroid (17-beta) dehydrogenase 11 | DHRS8 | -3,7 | -4,0 | -4,4 | -4,8 | -4,4 |
| Chromosome 5 open reading frame 30 | LOC90355 | -2,4 | -3,3 | -3,7 | -4,5 | -4,5 |
| Chitinase 3-like 1 (cartilage glycoprotein-39) | CHI3L1 | -3,2 | -4,5 | -4,9 | -3,8 | -4,5 |
| Shroom family member 2 | APXL | -4,9 | -4,3 | -3,9 | -6,3 | -4,5 |
| Transcribed locus | CLDN11 | -3,3 | -3,8 | -4,4 | -5,5 | -4,5 |
| regulator of G-protein signaling 4 | RGS4 | -3,9 | -4,6 | -4,8 | -4,0 | -4,6 |
| Plasminogen activator, urokinase | PLAU | -3,3 | -3,9 | -4,5 | -4,0 | -4,6 |
| Cyclin B1 | CCNB1 | -2,8 | -3,8 | -3,9 | -4,2 | -4,6 |
| Interferon-induced protein with tetratricopeptide repeats 1 | IFIT1 | -6,2 | -6,3 | -4,9 | -4,9 | -4,7 |
| Similar to hypothetical protein | LOC221091 | -3,9 | -3,8 | -4,6 | -4,5 | -4,7 |
| pleckstrin homology-like domain, family A, member 2 | PHLDA2 | -1,9 | -5,5 | -4,2 | -5,3 | -4,8 |
| Angiopoietin-like 1 | ANGPTL1 | -3,2 | -4,4 | -5,3 | -4,5 | -4,8 |
| Fibroblast activation protein, alpha | FAP | -2,6 | -2,9 | -3,3 | -6,7 | -4,9 |
| Tripartite motif-containing 22 | TRIM22 | -3,8 | -4,8 | -4,3 | -6,2 | -4,9 |
| PDZ binding kinase | PBK | -2,8 | -4,1 | -4,0 | -4,9 | -4,9 |
| V-fos FBJ murine osteosarcoma viral oncogene homolog | FOS | -7,9 | -5,9 | -5,8 | -8,0 | -5,0 |
| IkappaB-zeta | NFKBIZ | -1,7 | -4,2 | -5,1 | -5,4 | -5,0 |
| pleckstrin homology-like domain, family A, member 1 | PHLDA1 | -2,4 | -3,5 | -4,3 | -6,0 | -5,0 |
| Maternal embryonic leucine zipper kinase | MELK | -2,6 | -4,1 | -4,0 | -4,9 | -5,1 |
| Jun oncogene | JUN | -3,2 | -5,1 | -3,7 | -5,9 | -5,1 |
| Protein regulator of cytokinesis 1 | PRC1 | -2,4 | -3,6 | -3,6 | -6,0 | -5,2 |
| solute carrier family 14 (urea transporter), member 1 (Kidd blood group) | SLC14A1 | -5,3 | -5,1 | -6,1 | -4,7 | -5,2 |
| Transcribed locus | TCEAL7 | -1,9 | -3,4 | -3,6 | -7,3 | -5,2 |
| serpin peptidase inhibitor, clade B (ovalbumin), member 7 | SERPINB7 | -4,1 | -5,7 | -5,0 | -5,2 | -5,4 |
| Growth differentiation factor 5 | GDF5 | -6,6 | -5,7 | -5,3 | -5,5 | -5,4 |
| Transforming growth factor, beta receptor III | TGFBR3 | -2,0 | -3,7 | -5,9 | -5,5 | -5,5 |
| Frizzled homolog 2 (Drosophila) | FZD2 | -3,5 | -4,3 | -5,2 | -5,6 | -5,5 |
| Integrin, beta-like 1 (with EGF-like repeat domains) | ITGBL1 | -3,7 | -4,6 | -4,0 | -5,6 | -5,6 |
| ADAM metallopeptidase with thrombospondin type 1 motif, 5 (aggrecanase-2) | ADAMTS5 | -4,1 | -5,3 | -6,1 | -5,0 | -5,8 |
| Transcribed locus | MMP1 | -2,2 | -5,2 | -6,9 | -5,4 | -6,2 |
| thymosin beta 15a | TMSL8 | -3,8 | -6,0 | -4,9 | -5,8 | -6,2 |
| actin, alpha 2, smooth muscle, aorta | ACTA2 | -5,3 | -8,5 | -6,4 | -7,3 | -6,4 |
| ADAM metallopeptidase with thrombospondin type 1 motif, 1 | ADAMTS1 | -3,2 | -5,4 | -7,7 | -7,8 | -6,7 |
| Transcribed locus | COLEC12 | -3,6 | -4,8 | -5,9 | -13,3 | -7,0 |
| Potassium channel tetramerisation domain containing 4 | KCTD4 | -6,5 | -6,9 | -7,2 | -6,1 | -7,2 |
| Caveolin 1, caveolae protein, 22kDa | CAV1 | -3,3 | -5,5 | -5,7 | -11,0 | -7,7 |
| Neurotrimin | HNT | -6,4 | -8,6 | -8,5 | -6,9 | -7,9 |
| UDP-glucose ceramide glucosyltransferase | UGCG | -2,5 | -6,6 | -8,6 | -9,4 | -8,1 |
| Hyaluronan synthase 2 | HAS2 | -5,1 | -6,4 | -6,3 | -11,7 | -8,1 |
| chemokine (C-X-C motif) ligand 12; SDF1 | CXCL12 | -7,6 | -8,9 | -8,6 | -7,5 | -8,3 |
| Gremlin 1, cysteine knot superfamily, homolog (Xenopus laevis) | GREM1 | -7,1 | -11,1 | -7,7 | -12,7 | -9,0 |
| Insulin-like growth factor binding protein 3 | IGFBP3 | -2,1 | -5,1 | -8,2 | -13,2 | -9,0 |
| lysyl oxidase-like 1 | LOXL1 | -7,2 | -10,0 | -10,8 | -12,6 | -11,3 |
| Membrane metallo-endopeptidase | MME | -2,6 | -6,2 | -10,9 | -14,2 | -11,7 |
| Transmembrane protein 158 | TMEM158 | -1,9 | -10,6 | -16,9 | -16,9 | -15,2 |
| Polo-like kinase 2 (Drosophila) | PLK2 | -9,2 | -17,2 | -19,6 | -16,5 | -18,0 |
| vascular cell adhesion molecule 1 | VCAM1 | -5,6 | -32,3 | -38,5 | -43,1 | -49,9 |
