# Supplementary material for: Fetal Mesenchymal Stromal Cells Differentiating towards Chondrocytes Acquire a Gene Expression Profile Resembling Human Growth Plate Cartilage
Source: PLoS One. 2012 Nov 5;7(11):e44561. doi: 10.1371/journal.pone.0044561 (PMC3489884; doi:10.1371/journal.pone.0044561)
Supplement: Table S3 — List of top hits of differentially expressed genes of pre-pubertal GP with their fold change in expression compared to undifferentiated hfMSCs. (DOC) [file pone.0044561.s004.doc]

Table S3. List of genes that are ≥9 fold differentially expressed between pre-pubertal growth plate samples and undifferentiated hfMSCs.

| **GROWTH PLATE vs. hfMSC** | | |
| --- | --- | --- |
| **DESCRIPTION** | **GENE_SYMB** | **FOLD CHANGE** |
| collagen, type II, alpha 1 | COL2A1 | 103,08 |
| collagen, type IX, alpha 3 | COL9A3 | 92,60 |
| matrilin 1, cartilage matrix protein | MATN1 | 91,35 |
| fibroblast growth factor binding protein 2 | FGFBP2 | 83,17 |
| matrix metallopeptidase 9 | MMP9 | 82,26 |
| collagen, type IX, alpha 1 | COL9A1 | 72,87 |
| cartilage oligomeric matrix protein | COMP | 67,79 |
| secreted phosphoprotein 1 | SPP1 | 65,96 |
| pannexin 3 | PANX3 | 63,36 |
| stimulator of chondrogenesis 1 | SCRG1 | 59,80 |
| epiphycan | EPYC | 53,38 |
| leukocyte cell derived chemotaxin 1 | LECT1 | 53,37 |
| SPARC related modular calcium binding 2 | SMOC2 | 52,53 |
| chromosome 2 open reading frame 40 | C2orf40 | 49,99 |
| cytokine-like 1 | CYTL1 | 49,38 |
| hemoglobin, alpha 2; hemoglobin, alpha 1 | HBA2 | 48,85 |
| collagen, type XI, alpha 2 | COL11A2 | 48,75 |
| aggrecan | ACAN | 48,64 |
| frizzled-related protein | FRZB | 46,22 |
| hemoglobin, beta | HBB | 45,02 |
| fibromodulin | FMOD | 44,00 |
| matrilin 3 | MATN3 | 43,94 |
| procollagen C-endopeptidase enhancer 2 | PCOLCE2 | 41,26 |
| matrix Gla protein | MGP | 38,10 |
| chemokine (C-X-C motif) ligand 14 | CXCL14 | 37,86 |
| collagen, type X, alpha 1 | COL10A1 | 37,49 |
| vitrin | VIT | 36,46 |
| interleukin 17B | IL17B | 35,35 |
| WNT inhibitory factor 1 | WIF1 | 34,95 |
| acid phosphatase 5, tartrate resistant | ACP5 | 33,44 |
| collagen, type IX, alpha 2 | COL9A2 | 33,42 |
| S100 calcium binding protein A8 | S100A8 | 33,40 |
| serpin peptidase inhibitor | SERPINA5 | 33,28 |
| SRY (sex determining region Y)-box 8 | SOX8 | 32,77 |
| defensin, alpha 4, corticostatin | DEFA4 | 32,60 |
| S100 calcium binding protein B | S100B | 31,17 |
| GRAM domain containing 2 | GRAMD2 | 30,93 |
| retinol binding protein 4, plasma | RBP4 | 28,10 |
| lysophosphatidic acid receptor 4 | GPR23 | 27,99 |
| protocadherin 8 | PCDH8 | 27,99 |
| melanoma inhibitory activity | MIA | 27,59 |
| fibroblast growth factor receptor 3 | FGFR3 | 27,42 |
| cysteine-rich secretory protein LCCL domain containing 1 | CRISPLD1 | 27,27 |
| proline/arginine-rich end leucine-rich repeat protein | PRELP | 27,19 |
| matrilin 2 | MATN2 | 26,11 |
| integrin-binding sialoprotein | IBSP | 25,94 |
| carbonic anhydrase II | CA2 | 25,52 |
| chondroitin sulfate N-acetylgalactosaminyltransferase 1 | ChGn | 25,21 |
| protein tyrosine phosphatase, receptor-type, Z polypeptide 1 | PTPRZ1 | 24,25 |
| glycosyltransferase 25 domain containing 2 | GLT25D2 | 24,21 |
| nidogen 2 (osteonidogen) | NID2 | 23,53 |
| lipoprotein lipase | LPL | 23,48 |
| osteomodulin | OMD | 22,86 |
| FERM domain containing 4B | FRMD4B | 22,09 |
| FXYD domain containing ion transport regulator 6 | FXYD6 | 21,99 |
| chondroadherin | CHAD | 21,97 |
| C-type lectin domain family 3, member A | CLEC3A | 21,43 |
| zinc finger and BTB domain containing 20 | ZBTB20 | 20,18 |
| hCG2003663 | hCG_2003663 | 19,64 |
| alpha-2-macroglobulin | A2M | 19,58 |
| osteoglycin | OGN | 19,55 |
| carboxypeptidase X (M14 family), member 1 | CPXM1 | 19,50 |
| hemoglobin, delta | HBD | 19,38 |
| hypothetical gene supported by AK094963 | FLJ37644 | 19,17 |
| cysteine-rich secretory protein LCCL domain containing 2 | CRISPLD2 | 18,70 |
| platelet factor 4 | PF4 | 18,22 |
| collagen, type XV, alpha 1 | COL15A1 | 17,21 |
| zinc finger protein 385B | ZNF533 | 16,91 |
| serpin peptidase inhibitor, clade A (alpha-1 antiproteinase, antitrypsin), member 3 | SERPINA3 | 16,80 |
| integral membrane protein 2A | ITM2A | 16,72 |
| SRY (sex determining region Y)-box 9 | SOX9 | 16,69 |
| BMP and activin membrane-bound inhibitor homolog (Xenopus laevis) | BAMBI | 16,41 |
| calpain 6 | CAPN6 | 16,31 |
| X (inactive)-specific transcript (non-protein coding) | XIST | 16,11 |
| succinate dehydrogenase complex, subunit A, flavoprotein pseudogene 2 | SDHALP2 | 15,85 |
| DEP domain containing 6 | DEPDC6 | 15,80 |
| chordin-like 2 | CHRDL2 | 15,52 |
| collagen, type XVI, alpha 1 | COL16A1 | 15,48 |
| matrix metallopeptidase 13 (collagenase 3) | MMP13 | 15,43 |
| coagulation factor XIII, A1 polypeptide | F13A1 | 15,42 |
| sclerosteosis | SOST | 15,22 |
| hypothetical protein LOC646588 | LOC646588 | 15,06 |
| Boc homolog (mouse) | BOC | 14,87 |
| protease, serine, 35 | PRSS35 | 14,80 |
| transferrin | TF | 14,58 |
| serpin peptidase inhibitor, clade A (alpha-1 antiproteinase, antitrypsin), member 1 | SERPINA1 | 14,55 |
| clusterin | CLU | 14,06 |
| matrix extracellular phosphoglycoprotein | MEPE | 14,03 |
| upper zone of growth plate and cartilage matrix associated | C10orf49 | 13,60 |
| SPARC-like 1 (hevin) | SPARCL1 | 13,47 |
| WSC domain containing 2 | WSCD2 | 13,46 |
| RCSD domain containing 1 | RCSD1 | 13,36 |
| H19, imprinted maternally expressed transcript (non-protein coding) | H19 | 13,25 |
| cathepsin K | CTSK | 13,07 |
| lactotransferrin | LTF | 13,05 |
| hairy/enhancer-of-split related with YRPW motif 1 | HEY1 | 12,58 |
| gliomedin | GLDN | 12,50 |
| dishevelled associated activator of morphogenesis 1 | DAAM1 | 12,18 |
| adiponectin, C1Q and collagen domain containing | ADIPOQ | 11,97 |
| cysteine sulfinic acid decarboxylase | CSAD | 11,77 |
| matrix-remodelling associated 5 | MXRA5 | 11,70 |
| G protein-coupled receptor associated sorting protein 1 | GPRASP1 | 11,43 |
| fibulin 7 | FLJ37440 | 11,19 |
| protein phosphatase 1, regulatory (inhibitor) subunit 14C | PPP1R14C | 11,11 |
| solute carrier family 27 (fatty acid transporter), member 2 | SLC27A2 | 10,96 |
| multiple EGF-like-domains 10 | MEGF10 | 10,91 |
| dynein, cytoplasmic 1, intermediate chain 1 | DYNC1I1 | 10,86 |
| thyroid hormone receptor, beta (erythroblastic leukemia viral (v-erb-a) oncogene homolog 2, avian) | THRB | 10,85 |
| nuclear factor I/A | NFIA | 10,81 |
| lysosomal multispanning membrane protein 5 | LAPTM5 | 10,66 |
| chromosome 3 open reading frame 70 | LOC285382 | 10,63 |
| protein kinase C, zeta | PRKCZ | 10,60 |
| FAT tumor suppressor homolog 3 (Drosophila) | FAT3 | 10,48 |
| parathyroid hormone 1 receptor | PTHR1 | 10,44 |
| SERTA domain containing 4 | SERTAD4 | 10,35 |
| collagen, type XI, alpha 1 | COL11A1 | 10,27 |
| acid phosphatase-like 2 | ACPL2 | 10,12 |
| heparan sulfate (glucosamine) 3-O-sulfotransferase 3A1 | HS3ST3A1 | 10,08 |
| TAR DNA binding protein | TARDBP | 10,05 |
| C-type lectin domain family 4, member A | CLEC4A | 10,02 |
| EF-hand domain family, member D1 | EFHD1 | 10,01 |
| WNT1 inducible signaling pathway protein 3 | WISP3 | 9,97 |
| LIM and calponin homology domains 1 | LIMCH1 | 9,92 |
| paraoxonase 3 | PON3 | 9,92 |
| TRAF2 and NCK interacting kinase | TNIK | 9,90 |
| Ras-related GTP binding D | RRAGD | 9,88 |
| carbohydrate (N-acetylglucosamine 6-O) sulfotransferase 6 | CHST6 | 9,79 |
| solute carrier family 13 (sodium-dependent citrate transporter), member 5 | SLC13A5 | 9,76 |
| 3'-phosphoadenosine 5'-phosphosulfate synthase 2 | PAPSS2 | 9,70 |
| macrophage stimulating 1 (hepatocyte growth factor-like) | MST1 | 9,70 |
| carcinoembryonic antigen-related cell adhesion molecule 8 | CEACAM8 | 9,60 |
| myosin VC | MYO5C | 9,59 |
| hypothetical protein LOC387763 | LOC387763 | 9,56 |
| NOL1/NOP2/Sun domain family, member 6 | NSUN6 | 9,54 |
| dermatopontin | DPT | 9,48 |
| PP12104 | LOC643008 | 9,41 |
| sarcospan (Kras oncogene-associated gene) | SSPN | 9,16 |
| transcription elongation factor A (SII)-like 2 | TCEAL2 | 9,08 |
| integrin, alpha 10 | ITGA10 | 9,04 |
| stathmin-like 2 | STMN2 | -9,01 |
| protein tyrosine phosphatase, receptor type, K | PTPRK | -9,03 |
| tumor necrosis factor, alpha-induced protein 8 | TNFAIP8 | -9,05 |
| basonuclin 1 | BNC1 | -9,07 |
| interferon-induced protein with tetratricopeptide repeats 1 | IFIT1 | -9,07 |
| coagulation factor III (thromboplastin, tissue factor) | F3 | -9,12 |
| serpin peptidase inhibitor, clade E (nexin, plasminogen activator inhibitor type 1), member 1 | SERPINE1 | -9,18 |
| regulator of calcineurin 2 | RCAN2 | -9,21 |
| solute carrier family 1 (glial high affinity glutamate transporter), member 3 | SLC1A3 | -9,24 |
| chromosome 12 open reading frame 75 | OCC-1 | -9,29 |
| brain expressed, X-linked 1 | BEX1 | -9,32 |
| FBJ murine osteosarcoma viral oncogene homolog B | FOSB | -9,33 |
| sprouty homolog 2 (Drosophila) | SPRY2 | -9,35 |
| spastic ataxia of Charlevoix-Saguenay (sacsin) | SACS | -9,35 |
| protease, serine, 23 | PRSS23 | -9,42 |
| v-fos FBJ murine osteosarcoma viral oncogene homolog | FOS | -9,50 |
| RGM domain family, member B | RGMB | -9,64 |
| transmembrane protein with EGF-like and two follistatin-like domains 2 | TMEFF2 | -9,82 |
| transmembrane protein 132B; hypothetical LOC121296 | TMEM132B | -9,96 |
| ribosomal protein S4, Y-linked 1 | RPS4Y1 | -10,05 |
| synaptotagmin XI | SYT11 | -10,17 |
| glycoprotein (transmembrane) nmb | GPNMB | -10,21 |
| myosin light chain kinase | MYLK | -10,29 |
| pleckstrin homology-like domain, family A, member 2 | PHLDA2 | -10,30 |
| G protein-coupled receptor, family C, group 5, member A | GPRC5A | -10,32 |
| GLI pathogenesis-related 1 | GLIPR1 | -10,38 |
| sulfide quinone reductase-like (yeast) | SQRDL | -10,40 |
| similar to growth arrest-specific 6; growth arrest-specific 6 | GAS6 | -10,59 |
| bradykinin receptor B2 | BDKRB2 | -10,67 |
| Niemann-Pick disease, type C1 | NPC1 | -10,72 |
| ubiquitin carboxyl-terminal esterase L1 (ubiquitin thiolesterase) | UCHL1 | -10,96 |
| TOX high mobility group box family member 2 | TOX2 | -10,98 |
| chitinase 3-like 1 (cartilage glycoprotein-39) | CHI3L1 | -11,03 |
| pentraxin-related gene, rapidly induced by IL-1 beta | PTX3 | -11,06 |
| pleckstrin homology-like domain, family A, member 1 | PHLDA1 | -11,17 |
| coiled-coil domain containing 112 | CCDC112 | -11,41 |
| ADP-ribosylation factor-like 4C | ARL4C | -11,47 |
| UDP-glucose ceramide glucosyltransferase | UGCG | -11,49 |
| chromosome 15 open reading frame 48 | C15orf48 | -11,58 |
| brain-derived neurotrophic factor | BDNF | -11,67 |
| enolase 2 (gamma, neuronal) | ENO2 | -11,73 |
| guanylate binding protein 1, interferon-inducible, 67kDa | GBP1 | -11,77 |
| NAD(P)H dehydrogenase, quinone 1 | NQO1 | -12,07 |
| BCL2-associated athanogene 2 | BAG2 | -12,21 |
| insulin-like growth factor binding protein 4 | IGFBP4 | -12,23 |
| transgelin | TAGLN | -12,68 |
| regulator of G-protein signaling 4 | RGS4 | -12,84 |
| immediate early response 3 | IER3 | -12,86 |
| transmembrane protein 155 | TMEM155 | -13,01 |
| transmembrane protein 158 | TMEM158 | -13,27 |
| mannosidase, alpha, class 1A, member 1 | MAN1A1 | -13,38 |
| neuropilin (NRP) and tolloid (TLL)-like 2 | NETO2 | -13,55 |
|  | NA | -13,61 |
| follistatin-like 5 | FSTL5 | -13,66 |
| netrin 4 | NTN4 | -13,75 |
| Rho family GTPase 3 | RND3 | -14,22 |
| secretogranin II (chromogranin C) | SCG2 | -14,33 |
| frizzled homolog 2 (Drosophila) | FZD2 | -14,41 |
| cadherin 13, H-cadherin (heart) | CDH13 | -14,50 |
| ankyrin repeat domain 29 | ANKRD29 | -14,53 |
| major vault protein | MVP | -14,55 |
| pyruvate dehyrogenase phosphatase catalytic subunit 1 | PPM2C | -14,56 |
| claudin 11 | CLDN11 | -14,62 |
| contactin 3 (plasmacytoma associated) | CNTN3 | -14,64 |
| alpha-kinase 2 | ALPK2 | -15,25 |
|  | LOC728177 | -15,69 |
| hyaluronan synthase 2 | HAS2 | -15,78 |
| serpin peptidase inhibitor, clade B (ovalbumin), member 7 | SERPINB7 | -15,96 |
| sodium channel, voltage-gated, type III, alpha subunit | SCN3A | -16,14 |
| polo-like kinase 2 (Drosophila) | PLK2 | -16,34 |
| SRY (sex determining region Y)-box 11 | SOX11 | -16,37 |
| proteasome (prosome, macropain) subunit, beta type, 9 (large multifunctional peptidase 2) | PSMB9 | -16,48 |
| gremlin 1, cysteine knot superfamily, homolog (Xenopus laevis) | GREM1 | -16,84 |
| cornichon homolog 3 (Drosophila) | CNIH3 | -17,02 |
| fibroblast activation protein, alpha | FAP | -17,26 |
| vestigial like 3 (Drosophila) | VGLL3 | -17,63 |
| four and a half LIM domains 2 | FHL2 | -17,74 |
| ADAM metallopeptidase with thrombospondin type 1 motif, 1 | ADAMTS1 | -17,87 |
| vascular endothelial growth factor C | VEGFC | -18,16 |
| phorbol-12-myristate-13-acetate-induced protein 1 | PMAIP1 | -18,23 |
| ring finger protein 182 | RNF182 | -18,52 |
| similar to WDNM1-like protein | LOC645638 | -18,70 |
| transmembrane protein 200A | KIAA1913 | -19,36 |
| chromosome 4 open reading frame 49 | OSAP | -19,45 |
| brain abundant, membrane attached signal protein 1 | BASP1 | -19,68 |
| thymosin beta 15a; thymosin beta 15B | TMSL8 | -20,05 |
| actin, alpha 2, smooth muscle, aorta | ACTA2 | -20,15 |
| fibrillin 2 | FBN2 | -20,39 |
| membrane metallo-endopeptidase | MME | -20,66 |
| hypothetical LOC653631; hypothetical LOC646050; hypothetical LOC646890; axin interactor, dorsalization associated | C1orf80 | -21,41 |
| syncoilin, intermediate filament protein | SYNC1 | -22,30 |
| insulin-like growth factor binding protein 3 | IGFBP3 | -22,94 |
| cholesterol 25-hydroxylase | CH25H | -23,16 |
| insulin-like growth factor 2 mRNA binding protein 3 | IGF2BP3 | -23,51 |
| LRRN4 C-terminal like | LOC221091 | -24,12 |
| periostin, osteoblast specific factor | POSTN | -25,46 |
| DNA-damage regulated autophagy modulator 1 | DRAM | -27,47 |
| matrix metallopeptidase 1 (interstitial collagenase) | MMP1 | -28,53 |
| carboxypeptidase A4 | CPA4 | -31,74 |
| neurotrimin | HNT | -34,63 |
| transcription elongation factor A (SII)-like 7 | TCEAL7 | -35,48 |
| lysyl oxidase-like 1 | LOXL1 | -39,62 |
| calmegin | CLGN | -41,21 |
